# Psychological Interventions to Improve Elite Athlete Mental Wellbeing: A Systematic Review and Meta-analysis

**DOI:** 10.1007/s40279-024-02173-3

**Published:** 2025-01-15

**Authors:** Wei Wang, Matthew J. Schweickle, Emily R. Arnold, Stewart A. Vella

**Affiliations:** 1 School of Psychology, University of Wollongong, Wollongong, NSW 2500 Australia; 2 Global Alliance for Mental Health and Sport, University of Wollongong, Wollongong, Australia

## Abstract

**Background:**

Mental wellbeing, one continuum alongside mental illness in a dual-continua mental health model, has attracted less attention compared with substantial studies concerning mental illness amongst elite athletes. Notably, the promotion and protection of mental wellbeing contribute to not only a positive status of flourishing but also a reduction in the future risk of mental illness, which can potentially facilitate a status of complete mental health. Despite the critical role of wellbeing promotion and protection, there are limited evidence-based strategies to design and implement wellbeing interventions in elite athletes.

**Objective:**

This review aims to identify the different types of interventions to improve mental wellbeing amongst elite athletes and meta-analyse their effects. Further, the study aims to narratively identify the factors affecting implementation success in elite athletes.

**Methods:**

Peer-reviewed articles were systematically searched through five electronic databases (SPORTDiscus, PsycINFO, PSYCArticles, Academic Search Complete and MEDLINE) in July 2023 and updated in May 2024. To enrich the overall findings and reduce the risk of publication bias, grey literature was also included in this review. Dissertations and theses were the main foci and were searched in September 2023 and updated in May 2024. Standardised mean differences (SMDs) with 95% confidence intervals (CIs) were calculated to estimate the effects of the different types of interventions on mental wellbeing. Further, a narrative synthesis under the Consolidated Framework for Implementation Research was conducted to identify the potential factors that influenced the implementation success.

**Results:**

A total of 27 studies were found from peer-reviewed and grey literature, of which 15 studies were subject to meta-analyses. Results indicated that psychological skills training (SMD = 0.78, 95% CI 0.24, 1.32), third-wave interventions (SMD = 0.32, 95% CI 0.01, 0.63) and positive psychology interventions (SMD = 0.58, 95% CI 0.31, 0.85) were all potentially effective in improving overall wellbeing amongst elite athletes. However, when quasi-experimental studies in the third-wave interventions were removed for the sensitivity analysis, the effect was no longer significant. Further, 11 facilitators and 3 barriers to implementation success were identified, of which adaptability, coach and teammate support, and instructor’s capacity to connect with athletes were the key facilitators, and busy schedules and complex intervention contents were the main barriers.

**Conclusions:**

This review indicated that psychological skills training, third-wave interventions and positive psychology interventions could be potentially effective for elite athlete mental wellbeing, but more studies with robust experimental designs are needed in future to increase confidence in the favourable results. Moreover, future researchers and practitioners need to be aware of context-specific implementation facilitators and barriers, optimising elite athletes’ engagement and uptake of interventions.

**PROSPERO:**

CRD42023437986.

**Supplementary Information:**

The online version contains supplementary material available at 10.1007/s40279-024-02173-3.

## Key Points

**Table:** 

Psychological skills training, third-wave interventions and positive psychology interventions showed statistically significant effects on overall mental wellbeing in elite athletes.
The effect of third-wave interventions was no longer significant when quasi-experimental studies were removed, which indicated that the effect was not stable and future research with robust experimental designs is needed.
Adapting interventions, coach and teammate support, and instructor’s capacity to connect with athletes were the key facilitators for implementation success; busy schedules and complex intervention contents were two main barriers to athletes’ participation.

## Introduction

It is well known that participation in physical activity and sports can promote positive experiences and mental health across different populations [1]. However, competing in elite-level sports may detrimentally affect mental health owing to a diversity of context-specific stressors (e.g. lack of financial support, pressure of training full time and travelling long distances [2]). In the past decade, therefore, there has been a burgeoning interest in elite athlete mental health, but the focus has predominantly been on mental illness [3]. On the basis of a dual-continuum mental health model, however, mental health represents two different continua of mental illness and mental wellbeing [4]. In a scoping review of mental health in elite athletes (1998–2018), Kuettel and Larsen [3] noted that the vast majority of studies (around 86%) have primarily investigated mental illness such as depression and anxiety disorders. This predominant focus on mental illness has indirectly assumed that elite athletes are mentally healthy if they do not experience mental illness. However, the dual-continua model suggested that being mentally healthy represents not only the absence of mental illness but also the presence of good wellbeing (i.e. flourishing), and one study has shown that only about one-fifth of adults who did not have a common mental disorder (depression) experienced flourishing [4]. This model has been supported by several international mental health position statements in sport [5–7], and the critical role of moving beyond pathology was recently proposed in an evidence-informed mental health framework in elite sport [8].

In the Keyes’ mental health model, mental wellbeing is a combination of emotional, psychological and social wellbeing [4]. The dimension of emotional wellbeing was derived from hedonism, which emphasises the critical role of feeling good, and its indicators include positive affect and satisfaction [9]. Psychological wellbeing and social wellbeing were drawn from a eudaimonic perspective that focusses on doing well [9]. Specifically, psychological wellbeing refers to how an individual functions well in personal life and includes six aspects: self-acceptance, positive personal relations, autonomy, environmental mastery, purpose in life, and personal growth [10]. In contrast, social wellbeing focusses on positive functioning in society and includes five aspects: social acceptance, social actualisation, social contribution, social coherence and social integration [11]. The aforementioned dimensions of mental wellbeing align with Lundqvist’s [12] integrated conceptualisation of wellbeing in sport, which has been applied to elite orienteers [13] and elite para-athletes [14]. Furthermore, this multi-dimensional wellbeing can be distinguished into three levels on the basis of Keyes’ assessment (Mental Health Continuum-Short Form [15]), including flourishing (i.e. high wellbeing), moderate wellbeing and languishing (i.e. low wellbeing). Flourishing individuals have been found to experience high resilience, clear life goals and low helplessness [16], and these positive outcomes may also help elite athletes optimise their potentials in sports careers. One recent cross-sectional study reported that only 44.7% of elite athletes experienced flourishing [17], and thus, there is significant scope to support the promotion of mental wellbeing amongst elite athletes.

Notably, wellbeing promotion contributes to not only a positive status of flourishing, but also a reduction in the future risk of mental illness, which could increase the possibility of achieving complete mental health (i.e. the presence of flourishing and the absence of mental illness [16]). Data from a longitudinal study (1995–2005) showed that gains in mental wellbeing (e.g. from languishing to flourishing) predicted declines in mental illness, whilst reduction in mental wellbeing (e.g. from flourishing to languishing) predicted increases in mental illness [18]. This finding highlights the salience of mental wellbeing promotion (i.e. improving one’s wellbeing) and protection (i.e. maintaining one’s high wellbeing) in non-clinical populations. Recent meta-analyses indicated that the prevalence of mental disorders in elite athletes ranged from 19% (alcohol misuse) to 34% (anxiety/depression) [19]; that is, the majority of elite athletes are not mentally ill. Rather than dominantly focussing on athletes who have developed mental illness, the remaining, non-clinical individuals should also be targeted. As such, interventions to promote and protect mental wellbeing amongst those athletes are critical, as this could minimise their future risk of mental illness and lead them to a flourishing career in sport and life.

Unlike approaches to the management of mental disorders that have been increasingly investigated within elite athletes (e.g. [20–24]), sport practitioners have pointed out that actual strategies to improve athlete wellbeing are ambiguous [25]. Notably, mental wellbeing interventions are not the same as traditional approaches to treat mental illness. Treatment-based approaches focus on those who have developed mental illness, but wellbeing interventions target the majority whose mental wellbeing should be promoted and protected [9]. Outside the sport, mindfulness-based and positive psychological interventions were found to be particularly effective in improving mental wellbeing in the general population [26]. In sport, a meta-analysis showed that mindfulness-based programs demonstrated a large effect size for increasing psychological wellbeing in elite athletes [27]. However, there might be other types of interventions (e.g. psychological skills training) that are specifically acceptable and effective in sport [28]. As a result, systematically searching for and evaluating different types of interventions for elite athlete wellbeing could provide evidence-based recommendations for sport researchers and practitioners to design wellbeing programs in this context.

In addition to the development of wellbeing interventions amongst elite athletes, the role of implementation (i.e., a process of integrating an intervention into a particular setting [29]) is also of great significance. Durlak and Dupre [30] have found that the level of implementation affected the outcomes obtained in interventions, and they argued that designing a potentially effective intervention is only the first step, whilst transferring it into a real-world setting is a complicated process. The realm of elite sport, in particular, could be challenging to implement wellbeing interventions. For example, lack of buy-in from stakeholders such as coaches may hinder implementation. Wellbeing may not be an important interest in the elite sporting context where the primary purpose is to maximise performance. Further, elite athletes’ limited time and dynamic schedules may also negatively affect implementation success [31]. To the authors’ knowledge, however, no systematic reviews have specifically investigated the implementation of current psychology interventions amongst elite athletes. For instance, two recent systematic reviews within elite athlete populations have exclusively focussed on the intervention effectiveness on wellbeing (yoga interventions [32]; mindfulness-based programs [27]). The current research evidence can only inform sport psychology practitioners about which kinds of interventions might improve elite athlete wellbeing, but how those interventions can be properly implemented and attain expected outcomes in a real-world context is unknown. Accordingly, systematically exploring implementation issues in existing studies is critical and could facilitate the application of science-based wellbeing interventions in elite sport.

The study sought to fill the gaps and conduct a systematic review and meta-analysis to evaluate all extant interventions for elite athlete mental wellbeing. Specifically, the study was intended to systematically identify the different types of interventions to improve wellbeing amongst elite athletes. Second, the review aimed to meta-analyse the effects of those interventions. Third, amongst all identified interventions for wellbeing promotion, the study aimed to narratively synthesise the potential factors affecting their implementation success.

## Methods

### Protocol and Registration

The protocol was registered on PROSPERO (registration no. CRD42023437986). Some changes were made to the original PROSPERO protocol, and a modified version can be accessed at https://www.crd.york.ac.uk/prospero/display_record.php?ID=CRD42023437986. The modifications to the original protocol are reported in Online Resource 1. All methods of conducting and reporting this review followed the Preferred Reporting Items for Systematic Reviews and Meta-Analyses (PRISMA) guidelines [33]. The PRISMA checklist is reported in Online Resource 2.

### Eligibility Criteria

Studies were selected if they had full-text access and were written in English. With respect to types of studies, intervention studies that utilised randomised controlled, quasi-experimental, qualitative or mixed methods were included. Case studies were included if they used qualitative and/or quantitative methods, and this may help enrich the types of interventions and identify specific implementation facilitators and barriers amongst individual athletes. Further, interventions needed to be psychological interventions (i.e. psychological activities aim to improve mental health through intervening with regards to emotional states and behaviours [26]), whereas other types of interventions (e.g. nutritional interventions and physical training) were excluded. There were no limits regarding intervention duration. In terms of outcomes, studies needed to quantitatively and/or qualitatively report at least one of the following wellbeing outcomes: emotional wellbeing, psychological wellbeing, social wellbeing or integrated wellbeing. The definitions of wellbeing categories were based on Keyes’ [9] conceptualisation of mental wellbeing (Table 1). Wellbeing could be the primary or secondary outcome. Identified themes regarding wellbeing outcomes in qualitative studies need to align with the wellbeing categories in this review. Lundqvist [12] suggested that wellbeing in athletes should differentiate between the global level (e.g. life satisfaction) and the contextual sport level (e.g. sport satisfaction), and this review exclusively focussed on global wellbeing owing to limited valid and reliable measures to assess athletes’ wellbeing in sport [12, 34]. Studies were excluded if only reporting implementation factors (i.e. facilitators, barriers) but not reporting any wellbeing outcomes.

**Table 1:** Definitions of wellbeing categories

Wellbeing category	Definition [9]
Emotional wellbeing	Individuals’ positive feelings about life including positive affect and satisfaction
Psychological wellbeing	Individuals’ positive functioning in personal life including self-acceptance, positive relations with others, personal growth, purpose in life, environmental mastery and autonomy
Social wellbeing	Individuals’ positive functioning in society such as social coherence, social actualisation, social integration, social acceptance and social contribution

With respect to the eligibility criteria of participants, given the limited reporting of athlete success and experience in the literature, Swann et al.’s [35] Model for Classifying the Validity of Expert Samples in Sport Psychology was not applicable to define elite athletes in this review. However, following the standard of performance level (one aspect in their model), elite athletes in this review were operationalised as those who were competing at the university (i.e. student athletes needed to be in a university elite team such as National Collegiate Association Division I), national, international or Olympic levels. The level-based eligibility criteria have been widely used in other systematic reviews of elite athletes [22, 27, 36]. Studies were excluded if their participants were below 18 years old or if they used a mixed sample of athletes including any athletes below 18 years old. There are biopsychosocial differences between children and adults, meaning that the results from the different groups cannot be validly analysed together. Studies that had a mixed sample of elite and non-elite athletes without reporting group findings separately were excluded to reduce the heterogeneity of the data.

### Information Sources and Search Strategy

To conduct a comprehensive search, both peer-reviewed literature and grey literature were targeted. The search strategies are available in Online Resource 3.

#### Peer-Reviewed Literature

Five electronic databases were used to search for peer-reviewed literature: SPORTDiscus, PsycINFO, PSYCArticles, Academic Search Complete and MEDLINE. The primary search was conducted on 25 July 2023, followed by an update on 3 May 2024 (only including studies published after the primary search date). The first author collaboratively developed the initial search terms with the second and fourth authors, who have published several reviews regarding mental health in sport. Two previous systematic reviews about elite athlete mental health provided the basis on which initial terms were developed [27, 36]. The first author conducted several rounds of trials in the EBSCOhost database and refined the potential search string with other authors. The final terms were: (intervention* OR program* OR workshop* OR control* OR trial*) AND (wellbeing OR well-being OR wellness OR flourishing OR affect OR satisfaction OR happiness OR “mental health”) AND (athlet* OR player* OR Para-athlet*). Furthermore, manual search is recommended as a supplementary approach to an electronic search [37], and the first author hand-searched the included studies from relevant systematic reviews [27, 32, 38–40] (conducted alongside the primary search) and one scoping review [41] (conducted alongside the updated search). The first author also manually searched a list of all citations in each included study (conducted after the full-text review).

#### Grey Literature

Grey literature can be broadly described as multiple documents that are not published in a peer-reviewed academic journal [42]. The inclusion of grey literature can enrich the overall findings and reduce the risk of publication bias [43]. Dissertations and theses were the main foci of grey literature in this review. The search was conducted on 19 September 2023 through the ProQuest Dissertations & Theses Global and updated on 3 May 2024 (only including studies published after the first search date). Further, Google Scholar was also used to search for grey literature. Using the three keywords (intervention, wellbeing, athletes), the first 200 results (sorted by relevance) [44] on Google Scholar were included (conducted on 3 August 2023 and updated on 3 May 2024).

### Study Selection and Data Collection

Study selection in the primary search and updated search was completed in three phases through Covidence software (http://www.covidence.org, 25 July 2023 and 3 May 2024). First, all identified articles were imported in Covidence following the automatic removal of duplicates. Second, the first author and third author independently screened each article at the abstract and title level. Third, a full-text eligibility assessment was independently conducted by the first author and third author using a screening tool (Online Resource 4). A list of excluded studies is presented in Online Resource 5. The eligible full texts were double checked by the first author and were retained for data extraction if they met the inclusion criteria. All disagreements in the screening process were resolved via discussion between the two screeners.

An Excel spreadsheet was used for data extraction, including author(s) and year of publication, sport, country, intervention description (i.e. contents, primary aim, length), study design, sample characteristics (i.e. number of participants, mean age, female percent), wellbeing categories and measures, and relevant statistics to calculate individual studies’ standard mean difference and standard error. Unclear data were further clarified by contacting the corresponding authors of included studies. The first author extracted all data from eligible articles, and the third author checked 20% of the included studies to confirm the first author’s accuracy. The only one discrepancy between the two authors was wellbeing categories in a study, which indicated that data extracted by the first author were reliable.

### Critical Appraisal

The Mixed Methods Appraisal Tool (MMAT; version 2018) was used to appraise the methodological quality of the included studies [45]. The MMAT was developed specifically for different study designs and was suitable for this mixed studies review. All studies met the two MMAT qualifying criteria. The appraisal index was calculated by the number of ‘yes’ responses in each five quality criteria. For instance, the score was 20% when there was one ‘yes’ response and 100% for five ‘yes’ responses. The first author and third author conducted a critical appraisal on a random selection of 20% of the included studies (*k* = 6) to ensure consistency. All disagreements in the six studies were resolved, and all quality criteria were reviewed by the two authors. The first author then completed the critical appraisal for the remaining 21 studies.

### Strategies for Data Synthesis

Two approaches for data synthesis were adopted. First, meta-analyses were conducted to examine the effects of interventions reported within quantitative studies. Second, a narrative synthesis was employed to identify the potential factors that influenced the implementation of all included studies.

#### Meta-analysis

Meta-analyses were performed in JASP version 0.18.1 [46]. A random-effects model with the DerSimonian and Laird method [47] was used. Each study’s standardised mean difference (SMD, Cohen’s *d*) and standard error (SE) were imputed to JASP, where the pooled SMD was calculated to estimate summary effect sizes. The magnitude of effect sizes were interpreted as small (SMD = 0.2), medium (SMD = 0.5), and large (SMD = 0.8) [48]. The calculation process of individual studies’ SMD and SE is presented in Online Resource 6. Heterogeneity was assessed by *Q* statistics and *I*^2^ values. Q statistics were used to provide evidence that true effects vary. *I*^2^ values were used to estimate the level of heterogeneity with cut-offs of low (≤ 25%), moderate (25–75%) and high (≥ 75%) [49]. Egger’s test was used to examine evidence of publication bias [50]. Meta-regression analyses were not conducted in any meta-analysis owing to the risk of misleading results with fewer than ten studies [51]. Sensitivity analyses were conducted to assess whether the results differed between randomised controlled and quasi-experimental studies (e.g. non-randomised controlled trials).

#### Narrative Synthesis

A narrative synthesis (i.e. using words and text to synthesise findings [52]) was undertaken to identify the potential factors (facilitators and barriers) that influenced the current interventions’ implementation. This was conducted by the first author following Popay et al.’s [52] main phases of narrative synthesis. The Consolidated Framework for Implementation Research (CFIR) [53] was used to provide a conceptual framework that helped identify and summarise factors affecting implementation. The CFIR is composed of the following domains: intervention characteristics, inner and outer settings, characteristics of the individuals involved, and the process of implementation. Each domain includes related constructs; for example, intervention characteristics include adaptability and complexity. Following the approach that Kadu and Stolee [54] had used to identify facilitators and barriers to implementation, the reviewer read through all articles and identified the related ‘attributive statements’ (e.g. “The class session duration was shortened from the standard 150 min to promote high attendance and more time for self-practice” [55], p. 4), and coded them under the domains and constructs of the CFIR. These ‘attributive statements’ were from authors or participants (only in qualitative studies) and were often found in the procedure, results, and discussion section of an article. All statements are presented in Online Resource 7.

## Results

### Study Selection

**Fig. 1:**
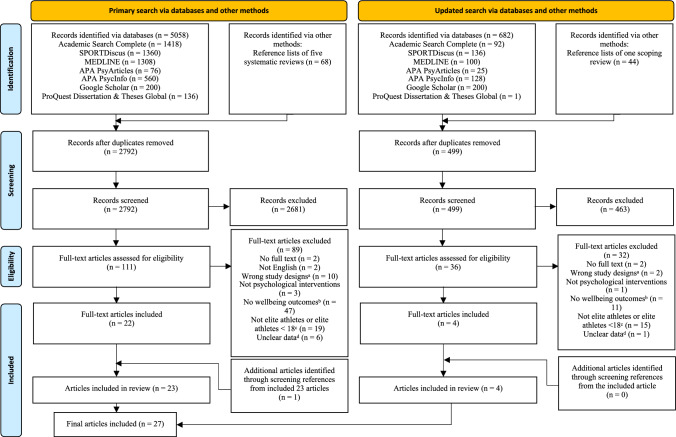
Preferred Reporting Items for Systematic Reviews and Meta-Analyses (PRISMA) flow diagram [33] of the eligibility process. ^a^Studies were not intervention research or did not use the following methods: randomised controlled, quasi-experimental, qualitative or mixed methods. ^b^Studies did not quantitatively or qualitatively report wellbeing outcomes defined in this review. ^c^Studies recruited athletes who were not at the elite level or elite athletes who were below 18 years old. ^d^Studies missed necessary data for eligibility assessment and their corresponding authors did not reply to our data request

The original search returned 5126 articles following the removal of 2334 duplicates. The remaining 2792 articles were screened for relevance, which resulted in 111 articles for full-text screening. Following the full-text stage, 22 studies were included. One additional study was identified through hand-screening references from the included full-text articles. Further, four studies were identified through the updated search. In total, 27 studies remained for data extraction. The PRISMA diagram of this process is provided in Fig. 1.

### Overview of Included Studies

Of the 27 studies that were included, 14 were quasi-experimental studies (non-randomised controlled trial, NRCT; no control group, pre/post), 4 were randomised controlled trials (RCTs), 4 were qualitative studies and 5 were mixed-method studies. There were 21 peer-reviewed articles, whereas the other 6 articles were sourced from grey literature. The mean age of elite athletes ranged from 18.1 to 32.5 years (six studies did not report mean age). The primary aim of those studies was to improve wellbeing (*k* = 20) or improve both wellbeing and performance (*k* = 7). The majority of interventions (88.9%) were conducted in Western countries (17 in North America, 4 in Europe, 3 in Australia, 2 in Asia and 1 in Africa). Further details of study characteristics are presented in Table 2.

**Table 2:** Study summary and critical appraisal

Author(s) and year	Intervention contents; primary aim; length	Sport; country	Study design	Sample characteristics	Wellbeing category	Measures	Critical appraisal
Ajilchi et al. [83]	7 sessions were based on the MAC approach; improving wellbeing; 7 weeks	Various individual sports; Iran	RCT 2-month follow-up	*n* = 45 Mage = 21.55 100.0% female	Psychological wellbeing	PWBS	40%
Baltzell and Akhtar [57]	12 sessions were based on the MMTS program; improving wellbeing; 6 weeks	Soccer, rowing; USA	NRCT	*n* = 42 Mage = unclear^a^ 100.0% female	Emotional wellbeing; psychological wellbeing	PANAS; SWLS; PWBS	40%
Baltzell et al. [63]	12 sessions were based on the MMTS program; improving wellbeing; 6 weeks	Soccer; USA	Qualitative	*n* = 7 Mage = unclear^a^ 100.0% female	Integrated wellbeing	N/A	80%
^*^Brent [69]	5 sessions consisted of topics such as relaxation, imagery, and self-talk; improving wellbeing and performance; 5 weeks	Unclear^a^; USA	NRCT 2-month follow- up	*n* = 30 Mage = 19.21 60.0% female	Emotional wellbeing	PANAS	60%
Chandler et al. [70]	10 sessions were based on the CMCL; improving wellbeing; 5 weeks	Football, basketball; USA	Mixed methods	*n* = 56 Mage = 18.3 17.9% female	Social wellbeing	PCQ	60%
Cote et al. [61]	6 modules were adapted from the MMTS program; improving wellbeing; unclear length^a^	Tennis; USA	Qualitative	*n* = 9 Mage = 21.3 44.4% female	Integrated wellbeing	N/A	80%
^*^Fallon [84]	Each session had three practices: physical postures, breath techniques, and meditation; improving wellbeing; unclear length^a^	Football; USA	Pre/post	*n* = 7 Mage = unclear^a^ 0.0% female	Emotional wellbeing	PANAS	40%
Fogaca [62]	5 sessions consisted of attributions and self-talk, arousal and anxiety regulation, mindfulness and acceptance, growth mindset, and integration; improving wellbeing and performance; 5 weeks	Various team and individual sports; USA	NRCT	*n* = 88 Mage = 19.8 51.0% female	Integrated wellbeing	WHOQOL	60%
Gabana et al. [58]	1 session consisted of didactic, gratitude writing, and discussion; improving wellbeing; 1 day	Various individual sports; USA	Pre/post 1-month follow-up	*n* = 51 Mage = 19.8 47.1% female	Emotional wellbeing; social wellbeing	SWLS; PASS-Q	60%
^*^Gavrilova [60]	12 sessions were based on TOPPS underlying positive psychology theory; improving wellbeing and performance; around 14 weeks	Team sport; USA	Pre/post 1- and 5-month follow-up	*n* = 1 Mage = unclear^a^ 100.0% female	Social wellbeing	SARI	80%
^*^Green [59]	4 sessions consisted of an introduction, goal orientation, personal meaning, and aims beyond self; improving wellbeing; 4 weeks	Track and Field; USA	Mixed methods	*n* = 1 Mage = unclear^a^ 0.0% female	Emotional wellbeing; psychological wellbeing	SWLS; MLQ	60%
Johnson [85]	3 sessions consisted of stress management and cognitive control, goal-setting skill, and relaxation/guided imagery; improving wellbeing; unclear length	Various team and individual sports; Sweden	RCT	*n* = 58 Mage = 23.7 10.3% female	Emotional wellbeing	MACL	20%
Jones et al. [55]	8 sessions were adapted from the MBSR program; improving wellbeing and performance; 9 weeks	Rowing; USA	NRCT	*n* = 27 Mage = 20.5 100.0% female	Psychological wellbeing	PWBS	20%
Laslett and Uphill [64]	2 online sessions consisted of a Therapeutic Letter to Self and Bull’s-eye Values Survey; improving wellbeing; around 4 weeks	Various team and individual sports; UK	Pre/post 2-week follow-up	*n* = 4 Mage = 23.25 50.0% female	Integrated wellbeing	SWEMWBS	40%
Laureano et al. [86]	6 sessions consisted of motivation, goal setting, time management, coping with injuries, emotion-focussed coping, getting into the ‘zone’; improving wellbeing; 2 weeks	Rugby; South Africa	NRCT	*n* = 41 Mage = 18.9 0.0% female	Emotional wellbeing	AFM-2	60%
^*^Leap [66]	10 sessions were based on the CMCL; improving wellbeing; 5 weeks	Football; USA	Mixed methods 3-week follow up	*n* = 15 Mage = 18.1 0.0% female	Psychological wellbeing; social wellbeing	N/A	40%
Macdougall et al. [14]	8 sessions were based on the MAC approach; improving wellbeing; 8 weeks	Various team and individual sports; Australia	RCT	n = 18 Mage = 32.5 72.2% female	Emotional wellbeing; psychological wellbeing; Social wellbeing	SPANE; SWLS; PWBS; SWBQ	60%
Miçoogullari and Ekmekci. [28]	48 sessions were based on the psychological skills training program: goal setting, imagery, self-talk, and arousal regulation; improving wellbeing; 16 weeks	Soccer; Turkey	Pre/post	*n* = 26 Mage = 26.69 0.0% female	Psychological wellbeing	PWBS	60%
Moesch et al. [87]	4 individual mindfulness sessions synchronised with web-based mindfulness practice; improving wellbeing; 8 weeks	Various team and individual sports; Sweden	Pre/post 10-week follow-up	*n* = 6 Mage = 26 66.7% female	Emotional wellbeing	WHO-5	60%
^*^Morton [68]	7 sessions consisted of signature strength, three good things, best possible self, counting one’s blessings, the gratitude letter, random acts of kindness, and choice; improving wellbeing; 7 weeks	Various team and individual sports; USA	Qualitative	*n* = 24 Mage = unclear^a^ 100.0% female	Integrated wellbeing	N/A	80%
Podlog et al. [88]	4 sessions consisted of injury education, attentional focus and distraction control, managing emotions, and pain management; improving wellbeing; 4 weeks	Various team and individual sports; USA	NRCT	*n* = 16 Mage = 19.94 75.0% female	Emotional wellbeing	PANAS	60%
Poulus et al. [89]	2 online sessions consisted of developing awareness of stressors and coping with stressors; improving wellbeing and performance; 5 weeks	Esports; Australia	Mixed methods 3-week follow-up	*n* = 5 Mage = 20.4 0.0% female	Integrated wellbeing	MHC-SF	80%
Reinebo et al. [67]	7 online sessions consisted of the elements of acceptance, values and mindfulness; improving wellbeing performance; 7 weeks	Ice hockey; Sweden	Qualitative	*n* = 4 Mage = 26 0.0% female	Psychological wellbeing	N/A	100%
Rooks et al. [78]	4 sessions were adapted from the MBSR program; improving wellbeing; 4 weeks	Football; USA	RCT	*n* = 100 Mage = 19.8 0.0% female	Emotional wellbeing	PANAS	20%
Stellefson et al. [90]	7 online sessions consisted of introduction, future vision, desired life outcomes, SMART goals, mental health management, physical well-being after athletic career, and personal action plans; improving wellbeing; 4–6 weeks	Various team and individual sports; USA	Pre/post	*n* = 14 Mage = 22.01 85.7% female	Psychological wellbeing	PWBS	60%
Van Ryswyk et al. [71]	2 sessions consisted of an initial sleep education session and a mid-program sleep education session; improving wellbeing and performance; 6 weeks	Football; Australia	Pre/post	*n* = 26 Mage = 23.7 3.8% female	Emotional wellbeing	POMS	20%
Vidic et al. [65]	10 sessions had the same elements: mindfulness education, guided calm-abiding meditation, and inquiry; improving wellbeing; 16 weeks	Basketball; USA	Mixed methods	*n* = 13 Mage = 19.85 100.0% female	Integrated wellbeing	N/A	60%

*AFM-2* Kammann and Flett’s (1983) Affectometer-2, *CMCL* Changing Minds, Changing Lives (10 sessions combine emotional regulation training, cognitive behavioural approaches, physical health information, social support, mindfulness and yoga practices), *MAC* Mindfulness Acceptance Commitment (8 sessions combine mindfulness practices, values exercises and acceptance techniques), *MACL* Mood Adjective Checklist, *MBSR* Mindfulness-Based Stress Reduction (8-week program consisted of mindfulness practices, psychoeducation and discussions), *MHC-SF* Mental Health Continuum-Short Form, *MLQ* Meaning in Life Questionnaire, *MMTS* Mindfulness Meditation Training for Sport (12 sessions comprising short mindfulness meditation practices and discussions), *N/A* not applicable*, NRCT* non randomised controlled trial, *PANAS* Positive and Negative Affect Schedule, *PASS-Q* Perceived Available Support in Sport Questionnaire, *PCQ* Perceived Cohesion Questionnaire*, POMS* profile of mood states, *Pre/post* pretest–posttest designs without control groups*, PWBS* Psychological Well-Being Scale, *RCT* randomised controlled trial, *SARI* Student Athlete Relationship Instrument, *SPANE* Scale of Positive and Negative Experience, *SWBQ* Social Well-Being Questionnaire, *SWEMWBS* Short Warwick–Edinburgh Mental Well-Being Scale, *SWLS* Satisfaction with Life Scale, *TOPPS* The Optimum Performance Program in Sports (12 sessions were based on positive psychology and Family Behaviour Therapy, aiming to optimise both performance and mental health), *WHO-5* World Health Organization Well-Being Index, *WHOQOL* World Health Organization–Quality of Life questionnaires

^*^Grey literature

^a^Data could not be found in the papers, and corresponding authors did not respond to data requests

### Results of Critical Appraisal

The average score of the critical appraisal across all 27 studies was 56%. Specifically, the mean scores of the qualitative studies, quantitative randomised controlled trials, quasi-experimental studies (i.e. NRCT and pre–post) and mixed method studies were 85%, 35%, 51% and 60%, respectively. The scores of the individual studies are presented in Table 2. All eligible studies contributed to the synthesis regardless of their appraisal outcomes, and this provided a complete overview of the evidence available for review [56]. The critical appraisal outcomes are available in Online Resource 8.

### Results of Types of Interventions

Overall, three types of interventions were identified to improve elite athlete wellbeing: psychological skills training (*k* = 5), third-wave interventions (*k* = 12) and positive psychology interventions (*k* = 5). The inclusion criteria were based on the different definitions of the three types of interventions given in this study (Table 3). The other types of interventions were integrated intervention of mental skills and mindfulness, coping effectiveness training for stressors, resilience training and sleep optimisation program.

**Table 3:** Definitions of types of interventions

Type of intervention	Definition	References
Psychological skills training (PST)	Systematic and consistent practice of mental skills to improve performance and self-satisfaction in sport competition, such as self-talk, goal setting, imagery and arousal regulation [91]	[28, 69, 85, 86, 88]
Third-wave interventions (TWIs)	Interventions based on the third-wave approaches that focus on changing relationships with inner experience (thoughts and feelings) rather than experience itself [73]. The common elements include mindfulness, acceptance, self-compassion and values [76]	[55, 57, 61, 63–65, 67, 78, 83, 84, 87, 92]
Positive psychology interventions (PPIs)	Interventions aim at increasing positive cognitions, emotions and behaviours as opposed to reducing mental health symptoms and disorders, which should be achieved through pathways consistent with the positive psychology theory [74]	[58–60, 68, 90]
Other interventions	Other psychological interventions	[62, 66, 70, 71, 89]

### Results of Meta-analyses

**Fig. 2:**
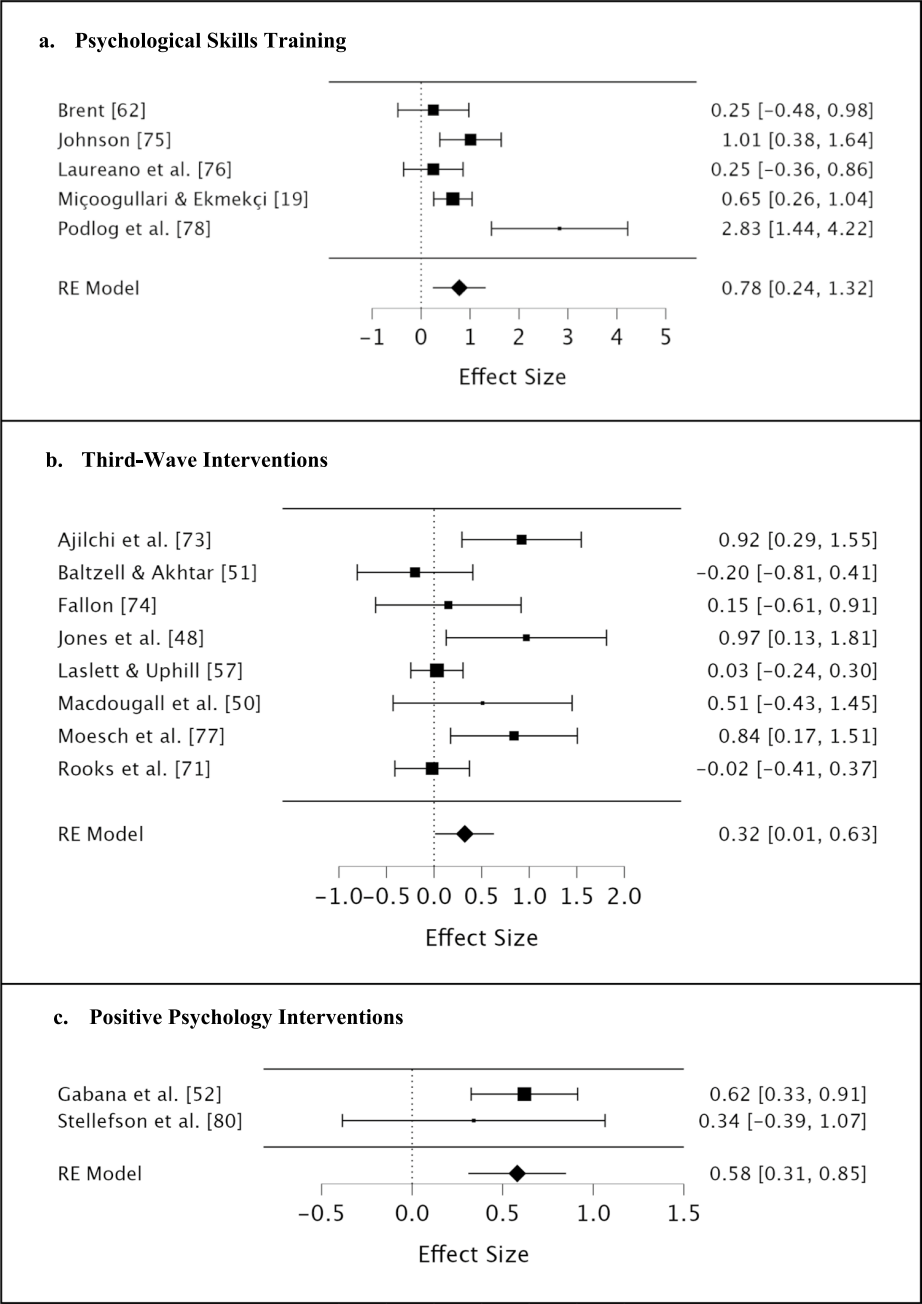
Forest plot for meta-analyses

Meta-analyses were performed separately for the three types of interventions. The results of meta-analyses for overall wellbeing (i.e. combining all wellbeing categories) are presented in Fig. 2.

#### Psychological Skills Training

Five quantitative studies were included in the meta-analyses. The first meta-analysis was used for overall wellbeing (*k*_studies_ = 5). There was a moderate statistically significant effect (standardised mean difference [SMD] = 0.78, 95% confidence interval [CI] 0.24–1.32, *p* = 0.005) (Fig. 2a). Heterogeneity was significant and moderate (*Q* = 13.52, *p* = 0.009; *I*^2^ = 70.41%, 95% CI 34.48–98.86%). Eggers’s test was significant (*Z* = 2.13, *p* = 0.033), which indicated potential publication bias. Further, another meta-analysis was specifically performed for emotional wellbeing (*k* = 4). There was a large statistically significant effect (SMD = 0.90, 95% CI 0.09–1.71, *p* = 0.030; *Q* = 13.51, *p* = 0.004; *I*^2^ = 77.79%, 95% CI 45.31–99.29%). Eggers’s test was significant (*Z* = 2.52, *p* = 0.012). Sensitivity analysis was not conducted owing to the small number of RCTs (*k* = 1).

#### Third-Wave Interventions

Eight quantitative studies were included in the meta-analyses. Macdougall et al. [14] separately measured emotional, psychological and social wellbeing, and the SMD was calculated by taking the mean effect size of the three categories. Baltzell and Akhtar’s [57] study was excluded owing to the unclear data on psychological wellbeing. The first meta-analysis was used for overall wellbeing (*k* = 8). There was a small statistically significant effect (SMD = 0.32, 95% CI 0.01–0.63, *p* = 0.042) (Fig. 2b). Heterogeneity was significant and moderate (*Q* = 16.59, *p* = 0.020; *I*^2^ = 57.81%, 95% CI 3.52–91.30%). Egger’s test was not significant (Z = 1.84, *p* = 0.066), indicating low potential publication bias. Sensitivity analysis was conducted by removing quasi-experimental studies; the remaining RCTs (*k* = 3) demonstrated a non-significant effect (SMD = 0.43, 95% CI − 0.22 to 1.08, *p* = 0.198). Heterogeneity was significant and moderate (*Q* = 6.48, *p* = 0.039; *I*^2^ = 69.13%, 95% CI 0–98.88%).

Further, the second and third meta-analyses were respectively used for emotional wellbeing (*k* = 5) and psychological wellbeing (*k* = 3). The effect of emotional wellbeing was not significant (SMD = 0.27, 95% CI − 0.16–0.70, *p* = 0.216; *Q* = 9.03, *p* = 0.060; *I*^2^ = 55.71%, 95% CI 0–95.58%). Sensitivity analysis was not conducted owing to the small number of RCTs (*k* = 1). Egger’s test was not significant (*Z* = 1.48, *p* = 0.139). The effect of psychological wellbeing was large and statistically significant (SMD = 0.82, 95% CI 0.38–1.27, *p* < 0.001; *Q* = 0.91, *p* = 0.633; *I*^2^ = 0%). Egger’s test was not significant (*Z* =  − 0.64, *p* = 0.525). Sensitivity analysis was conducted by removing quasi-experimental studies; the remaining RCTs (*k* = 2) demonstrated a statistically significant effect (SMD = 0.77, 95% CI 0.24–1.29, *p* = 0.004). Heterogeneity was not significant (*Q* = 0.75, *p* = 0.386; *I*^2^ = 0%).

#### Positive Psychology Interventions

Two quantitative studies (135 athletes) were included in the meta-analysis for overall wellbeing. Only the result of social wellbeing in Gabana et al.’s [58] study was included in the meta-analysis owing to the unclear data on emotional wellbeing. Two single-case studies with one participant were excluded [59, 60]. There was a moderate statistically significant effect (SMD = 0.58, 95% CI 0.31–0.85, *p* < 0.001) (Fig. 2c). Heterogeneity was not significant (*Q* = 0.49, *p* = 0.483; *I*^2^ = 0%). Egger’s test could not be run in JASP owing to the limited number of studies (*k* = 2). Sensitivity analysis was not conducted because there were only quasi-experimental studies.

### Results of Factors Affecting Implementation

The narrative synthesis consisted of 15 studies. The results of factors (i.e. facilitators and barriers) affecting implementation under the domains and constructs of the CFIR are presented in Table 4.

**Table 4:** Facilitators and barriers to implementation under the Consolidated Framework for Implementation Research (CFIR) domains and constructs [53]

Domain	Construct	Facilitator	Barrier
Intervention characteristics	Adaptability	Using language accessible to athletes [61] Incorporating sport psychological elements [57, 62] Shortening duration of each session [55] Applicability to sport and life [61, 63–67]	
	Complexity		Complex and unfamiliar intervention contents [61, 63, 65, 67, 69]
Individual characteristics	Personal attributes	Instructor’s capacity to connect with athletes [61, 63, 67, 68]	
Inner setting	Readiness for implementation	Coach support [63, 68] Available resources [55]	
	Implementation climate	Relaxing learning climate [61]	
Process	Engaging	Multiple in-session activities [59, 67] Between-session assignments [59] Teammate support [58, 63, 66, 69, 70]	
	Executing		Athletes’ busy schedules [61, 62, 65, 84]
	Reflecting and evaluating		Risk of bias [61]

#### Facilitators

Eleven facilitators were identified through 14 studies: 1 study was from psychological skills training (PST), 7 studies were from third-wave interventions (TWIs), 3 studies were positive psychology interventions (PPIs), 1 study used an integrated intervention of PST and TWIs and 2 studies were resilience interventions. The facilitators were explained under the seven constructs of the CFIR below.

*Adaptability.* Interventions that were tailored to the sport-specific context facilitated implementation success. First, Cote et al. [61] indicated the importance of using appropriate language (i.e. framing self-compassion as a catalyst of courage) to increase receptivity and skill acquisition. Second, two studies from TWIs and the other interventions incorporated PST techniques (e.g. self-talk and imagery), and this integration helped meet the needs and the buy-in of athletes [57, 62]. Third, intervention length could be adapted to fit elite athletes’ schedules, such as shortening the duration of each session, which could promote high attendance [55]. Fourth, the specific applicability to sport and life helped increase athletes’ interest and commitment to interventions [61, 63–67].

*Personal attributes.* Instructors’ capacity to connect with participants was vital for implementation success. For example, Baltzell et al. [63] reported that the instructor attended one intervention group’s match, which increased athletes’ trust and connection with the instructor. Further, in Morton’s [68] study, the instructor worked as a volleyball and gymnastics sport psychology consultant before intervention implementation with the two teams, and this was critical for the athletes’ buy-in and commitment to the program.

*Readiness for implementation.* This construct refers to tangible and immediate indicators of organisational commitment to implement an intervention [53]. Two studies reported that coach support (i.e. participating in some sessions) was an important facilitator for the implementation [63, 68]. Furthermore, available resources such as workbooks and audio recordings of practices could help increase athletes’ uptake of a complicated mindfulness program [55].

*Implementation climate.* A relaxing implementation climate helped facilitate participation and intervention success. Cote et al. [61] reported that the mindfulness learning environment helped them slow down and take a pause on their athletic and personal responsibilities.

*Engaging.* Multiple in-session activities helped increase athletes’ engagement and uptake of the program [59, 67]. For example, all athletes in an internet-based intervention reported that reading texts, doing audio exercises and watching videos were complementary and helpful to their engagement and learning [67]. Between-session assignments were also found to be a useful element to engage athletes in a four-session intervention [59]. Furthermore, delivering interventions with a group of athletes improved team cohesion and implementation success [58, 63, 66, 69, 70]. For instance, athletes in a group-based mindfulness intervention reported that meditating with their teammates facilitated team cohesion and their participation in the program [63].

#### Barriers

Several barriers were identified through seven studies: one study was from PST, five studies were from TWIs, and one study used an integrated intervention of PST and TWIs. The barriers were explained under the three constructs of the CFIR below.

*Complexity.* This construct refers to perceived difficulty of interventions [53]. Complex and unfamiliar intervention contents hindered implementation. Many studies in TWIs reported that athletes felt weird and found it difficult to sit and practise meditation [61, 63, 65]. Developing a direct connection between meditation practices and sport/life may be a strategy to solve this barrier [63]. Additionally, the athletes in a PST program reported that progressive muscle relaxation was difficult to practise on their own [69], and a recording of the exercise may support their independent practice.

*Executing.* Elite athletes’ busy schedules became a primary barrier in executing an intervention. For instance, Vidic et al. [65] had to adjust the frequency of sessions during athletes’ busy competitive season. Further, athletes in a mindfulness program reported that they lacked time to practise meditation consistently [61]. One solution for this barrier might be implementing interventions during the off-season or pre-season [62, 71].

*Reflecting and evaluating.* This construct refers to quantitative and qualitative feedback about the progress and quality of interventions [53]. The potential risk of bias hindered a critical evaluation. In Cote et al.’s [61] study, only 9 of the 23 athletes agreed to attend the post-intervention interviews, indicating a potential bias that participants who had more positive experience were more willing to provide feedback.

## Discussion

This systematic review and meta-analysis aimed to synthesise research regarding interventions for elite athlete mental wellbeing. Specifically, three main objectives were to: (a) identify the types of interventions used to enhance wellbeing amongst elite athletes, (b) meta-analyse the effects of interventions on wellbeing and (c) narratively synthesise the potential factors affecting implementation success. The three aims were achieved in the review and are discussed in turn.

### Types of Interventions

PST, TWIs and PPIs were the three main types of interventions to improve mental wellbeing amongst elite athletes, and they have been conceptually distinguished in this review. PST shares a purpose similar to that of traditional cognitive behavioural therapy and aims at controlling and changing one’s internal thoughts, emotions and behaviours [72]. Some basic techniques such as self-talk, imagery, goal setting, arousal regulation and thought stopping were reported in the included studies. By contrast, TWIs focus on shifting the relationships with inner experience rather than changing its contents [73]. Mindfulness, acceptance and values are the components of TWIs in this review. Further, with regard to PPIs, their core purpose is to increase positive feelings, cognitions and behaviours on the basis of the principles of positive psychology [74]. The main PPI elements in this review included gratitude, relationship enhancement, positive goals, signature strengths and personal meaning. Although PPIs may share some overlap with PST (e.g. goal setting) and TWIs (e.g. values), the review regarded it as an independent type of intervention that reflects the theoretical tradition of positive psychology.

### Effects of the Interventions

With respect to the results of meta-analyses, PST demonstrated a moderate statistically significant effect on overall wellbeing amongst elite athletes. Further, an additional meta-analysis was conducted for emotional wellbeing, which showed a large statistically significant effect size. Despite the favourable results, there remains some issues that researchers and practitioners need to be aware of. First, four of the five studies used quasi-experimental designs, and lack of rigorous research methods such as RCTs may decrease the confidence in the promising results. Second, whilst the heterogeneity was significant, meta-regression was not conducted owing to the small number of studies, thus the potential elements that may explain between-study variability remain unknown. For example, there were three different psychometric measures assessing emotional wellbeing, and this may become a reason for the significant heterogeneity. Third, despite the extensive search (e.g. including grey literature) in this review, there is still a possibility of publication bias as the Egger’s test was significant. Studies with non-significant results may not be published and were not identified by the search of grey literature. Fourth, there is a lack of theoretical mechanisms to explain the favourable effects of PST on mental wellbeing. PST was originally designed to enhance sport performance, and its theoretical foundation is that optimal performance occurs when athletes control their negative thoughts and emotions [75]. As such, there is substantial opportunity for researchers to conduct robust RCTs and to investigate the mechanisms of PST acting on mental wellbeing.

Similar to PST, TWIs had a small but statistically significant effect on overall mental wellbeing in elite athletes, which was consistent with another meta-analysis where it was found that TWIs demonstrated a moderate effect on mental wellbeing amongst children and adolescents [76]. However, sensitivity analysis showed that a non-significant effect when quasi-experimental studies were removed from the synthesis. As a result, researchers and practitioners need to interpret the promising result cautiously. Two additional meta-analyses in TWIs were conducted for two specific wellbeing categories. There was a large statistically significant effect on psychological wellbeing but a non-significant effect on emotional wellbeing. All studies in the meta-analyses were mindfulness-related interventions, but the results were not consistent with a mindfulness-to-meaning theory which proposed that the practice of mindfulness cultivates metacognitive awareness that transforms how people relate to their adverse events, and thus leads to increases in both positive affect (emotional wellbeing) and purpose in life (psychological wellbeing) [77]. One potential explanation for this inconsistency is that elite athletes experienced high demands during the intervention period and short-term mindfulness training was not able to increase their positive emotions. However, short mindfulness programs could protect against the decline in positive emotions if athletes have greater engagement in programs [78]. As such, optimising athletes’ participation in the implementation process may be helpful in improving the effectiveness of short programs (also discussed in the “Implementation” section below).

With regard to the results of PPIs, a moderate statistically significant effect was found on elite athlete overall wellbeing. The result was consistent with another meta-analysis where it reported that PPIs demonstrated a small but statistically significant effect on mental wellbeing amongst general populations [74]. Despite the promising finding, this meta-analysis only included two quasi-experimental studies owing to limited data, which suggests that more PPIs with robust experimental designs are needed to increase confidence in the evidence. Further, it is noteworthy that PPIs such as gratitude interventions may play an important role in elite athletes’ social wellbeing. For example, Gabana et al. [58] conducted a gratitude workshop amongst elite student athletes and found that their perceived social support was improved. Negative social relationships and lack of support have been found to be risk factors for elite athlete mental health [3], and therefore, there remains substantial room for researchers and practitioners to develop tailored PPIs (e.g. gratitude interventions) to improve social connection and wellbeing amongst elite athletes.

### Factors Affecting Implementation

Eleven facilitators and three barriers were identified through narrative synthesis. The primary facilitative factors were appropriate adaptability, instructor’s capacity to connect with athletes, coach and teammate support, and available resources; the main barriers were complex intervention contents and athletes’ busy schedules. Notably, the key facilitator of interventions adapted to the context and the barrier of time constraints were also identified in another two studies [54, 79].

#### Balancing Adaptability and Fidelity

On the basis of the findings of the narrative synthesis, adaptability plays an indispensable role in facilitating implementation, and yet, its relations with program fidelity need to be carefully considered. Given that elite athletes are in a specific context, the adaptation of an original program is inevitable to meet their unique needs. For example, the four facilitators of adaptability in this review included using appropriate language, incorporating sport psychology, shortening sessions and applying programs to sport and life. However, fidelity should not be compromised when adapting a program. Fidelity refers to the extent to which an intervention corresponds to an original program. Without fidelity, an intervention may lose some essential program features, and this can undermine the theoretical mechanisms of an evidence-based program and affect its effectiveness [30]. In balancing fidelity and adaptation, one may consider both the theoretically essential and flexible components of a program [80]. Core intervention elements should be inflexible to promote program fidelity, and other less essential ones can be adapted to achieve a good fit. For instance, when the Mindfulness-Based Stress Reduction program was tailored to athletes, Jones et al. [55] retained the essential components of a mindfulness-based program (e.g. intensive training in mindfulness practice) and, concomitantly, adapted the intervention length to better fit athletes’ context. Therefore, explicitly naming the essential and flexible components of intervention so as to balance fidelity and adaptability would be beneficial, which could in turn help promote both effectiveness and implementation success.

#### Optimising Participation When Delivering a Program

In addition to the appropriate adaptability, optimising elite athletes’ participation is also important for implementation success. There were three primary components affecting athletes’ engagement when delivering a program: time, people and resources. First, this review found that elite athletes’ busy schedule was regarded as a barrier to engagement, and thus choosing an appropriate time to deliver an intervention is important. Two studies in this review suggested that conducting interventions during the off-season or pre-season period would facilitate implementation [62, 71]. Second, instructors, coaches and teammates could increase athletes’ commitment to interventions. Instructors need to have the capacity to connect with elite athletes, who might have less trust in people outside their sports, and attending their matches could establish trust and rapport [63]. Several studies also mentioned that coach presence [63, 68] and teammate support [58, 63, 69] improved athletes’ interest and adherence to the interventions, and these two facilitative elements can be particularly important in elite sport. Third, learning resources might help promote athletes’ continuous engagement, especially for complicated and unfamiliar interventions. Specifically, recorded audio files in PST and TWIs could support athletes’ independent home practice [55, 69].

## Limitations

This review is not without its limitations. First, following Keyes’ [9] definitions of mental wellbeing, the review only included studies that assessed emotional wellbeing (i.e. positive affect, satisfaction with life), psychological wellbeing and social wellbeing on a global level. There remains considerable conceptual and methodological variability of wellbeing in sport [12], and studies that measured other wellbeing outcomes (e.g. negative affect) and focussed on a sport-specific level were excluded. Second, the overall study sample was dominated by elite athletes in Western countries (88.9%), thus the results may not be generalisable to athletes who are exposed to other cultures (e.g. in Asia and the Middle East). Third, the Mixed Methods Appraisal Tool was used to appraise the methodological quality of the included studies, given the range of study designs included in the review. However, this tool only has five quality criteria for each study design, and as such, the results of critical appraisal in this review need to be interpreted with caution.

In addition, with respect to the meta-analyses, this review included both RCTs and quasi-experimental studies, and the large number of quasi-experimental studies may have decreased the quality of evidence. For example, sensitivity analysis was conducted in TWIs, and the remaining RCTs demonstrated a non-significant effect when removing quasi-experimental studies. Further, whilst heterogeneity was significant and moderate in the results of meta-analyses in PST and TWIs, meta-regression analyses were not conducted owing to the risk of misleading results with fewer than ten studies [51]. This makes it difficult to investigate the impacts of experimental differences on intervention effects. For example, the number of sessions in TWIs varied between 2 and 12 sessions and the length of interventions varied between 4 and 9 weeks, which may influence the intervention effects. However, a meta-regression analysis of randomised controlled trials (*k* = 207) reported that the number of sessions and intervention length were not significant moderators for the effectiveness of mindfulness-based programs on mental health outcomes (i.e. depression, anxiety and stress) [81]. Lastly, there were limited findings regarding the factors affecting implementation success. Only around half of the included studies (55.6%) contributed to the narrative synthesis, and the majority of studies were from TWIs and PPIs. There remains substantial room for future interventions to improve the evaluation of the implementation process.

## Recommendations

Psychological interventions to improve mental wellbeing amongst non-clinical elite athlete populations play a significant role in the promotion of flourishing and the reduction of future risk of mental illness. This research area, however, has been overlooked in elite sport. We conducted a comprehensive systematic review that aimed to provide valuable recommendations in this area. First, three types of interventions (i.e. PST, TWIs and PPIs) were identified to improve elite athlete wellbeing, and researchers may consider investigating their mechanisms of change in this population. Seven studies in this review have mixed the intervention aims of performance and wellbeing (Table 2), which may lead to the ambiguity about how those interventions can specifically improve athlete wellbeing. This recommendation aligns with Breslin et al.’s consensus statement where they recommended that the development of mental health interventions in sport should be underpinned by appropriate theories and models, such that the theoretically driven mechanisms of change could facilitate the effectiveness of the interventions [6]. In addition, PPIs (e.g. gratitude interventions) have theoretically driven mechanisms of change [82] and have demonstrated significant effects on the promotion of wellbeing outside the sport [26], and yet, there are currently limited PPIs within elite athlete populations. Researchers can adapt those evidence-based PPIs to the elite sporting context and evaluate their feasibility and acceptability.

Furthermore, owing to the limited numbers of RCTs (*k* = 4) in this review, researchers in elite sport may consider adopting robust experimental designs such as RCTs to further assess the effectiveness of the three types of interventions on athlete wellbeing. More robust RCTs can provide further recommendations concerning whether those interventions would be worthy of being delivered in a large scale-up to help non-clinical elite athlete populations achieve a higher level of mental health. Lastly, conducting RCTs in this context could be challenging, and researchers need to be mindful of specific implementation facilitators and barriers identified in this review, as well as other factors that may relate to individual differences (e.g. cognitive and behavioural differences amongst individuals) and cultural differences (e.g., Western and Asian cultures). Studies in this review have dominantly focussed on the intervention outcomes but overlook the importance of the implementation process. Improving the evaluation of the implementation process in future research may provide valuable implications with sport practitioners when distributing those evidence-based interventions on a large scale-up.

## Conclusions

The systematic review and meta-analysis showed that PST, TWIs and PPIs were the three main types of interventions for mental wellbeing amongst elite athletes, and they were all potentially effective. Furthermore, 11 facilitators of and 3 barriers to implementation were found through narrative synthesis. Specifically, adaptability was a primary facilitator for intervention success, but fidelity needs to be balanced when tailoring an intervention to elite sport. Busy schedules amongst athletes and complex intervention contents were two key barriers to athletes’ participation, and off-season or pre-season delivery, instructor competence, coach and teammate support, and available learning recourses can help promote athletes’ engagement and commitment to interventions. In the future, researchers and practitioners need to focus on not only the design of potentially effective interventions for elite athletes but also the implementation process in this specific context.

## Supplementary Information

Below is the link to the electronic supplementary material.

Supplementary file1 (DOCX 42 KB)

Supplementary file2 (DOCX 43 KB)

Supplementary file3 (DOCX 59 KB)

Supplementary file4 (DOCX 33 KB)

Supplementary file5 (DOCX 37 KB)

Supplementary file6 (DOCX 84 KB)

Supplementary file7 (DOCX 113 KB)

Supplementary file8 (DOCX 38 KB)

## Declarations

### Availability of data and material

All data have been included in the article or included as supplementary information.
