# Supplementary material for: Psychological Interventions to Improve Elite Athlete Mental Wellbeing: A Systematic Review and Meta-analysis
Source: Sports Med. 2025 Jan 15;55(4):877–97. doi: 10.1007/s40279-024-02173-3 (PMC12011916; doi:10.1007/s40279-024-02173-3)
Supplement: Supplementary file 1 — Supplementary file1 (DOCX 42 KB) [file 40279_2024_2173_MOESM1_ESM.docx]

**Supplementary information. Online Resource 1.**

*Article:* Psychological Interventions to Improve Elite Athlete Mental Wellbeing: A Systematic Review and Meta-Analysis

*Journal:* Sports Medicine

*Authors:* Wei Wang, Matthew J. Schweickle, Emily Arnold, Stewart A Vella

*Corresponding author:* Wei Wang, School of Psychology, University of Wollongong, Wollongong, New South Wales, 2500, Australia. Email: ww862@uowmail.edu.au

**Amendments to the original protocol (The original and modified versions of the protocol can be access at** <https://www.crd.york.ac.uk/prospero/display_record.php?ID=CRD42023437986>)

|  | Amendment | Reason |
| --- | --- | --- |
| 1 | One research question has been amended.  The original question was: “What are the facilitators and barriers that may influence the *effects* of current interventions?” The current question is: “What are the facilitators and barriers that may influence the *implementation success* of interventions among elite athletes?” | The factors influencing the effects of intervention may be difficult to be identified through a systematic review. However, the factors influencing implementation success may be identified through a narrative synthesis. |
| 2 | Two search strategies have been added.   1. Searching grey literature (i.e., dissertations and theses) via the ProQuest Dissertations & Theses Global and Google Scholar. 2. Manually searching relevant systematic reviews and reference lists of all included studies. | These amendments can help reduce the risk of publication bias and facilitate a comprehensive search. |
| 3 | The exclusion criteria of participants have been amended.  Young elite athletes (below 18 years), retired elite athletes, and mixed samples of elite and non-elite athletes have been excluded. | Excluding young athletes, retired athletes, and mixed samples of elite and non-elite athletes can reduce the heterogeneity of the data. For example, children and adults have biopsychosocial differences, and the intervention results from the two groups may not be validly analysed together. |
| 4 | The eligibility criteria for intervention outcomes have been amended.  The original protocol accepted the studies that assessed mental wellbeing on a global level or a sport-specific level. The modified protocol only accepts those studies assessing mental wellbeing on a global level. | This amendment may help increase the validity of the results of meta-analyses in the review because there are limited valid and reliable measures to assess athletes’ wellbeing in sport [1,2]. |
| 5 | The risk of bias assessment tool has been amended.  The original tool was the Joanna Briggs Institute’s checklists. The current tool is The Mixed Methods Appraisal Tool. | The Mixed Methods Appraisal Tool [3] was developed specifically for different study designs and is more suitable for this mixed studies review than the Joanna Briggs Institute’s checklists. |

**References:**

1. Giles S, Fletcher D, Arnold R, Ashfield A, Harrison J. Measuring Well-Being in Sport Performers: Where are We Now and How do we Progress? Sports Medicine. 2020;50:1255–70.

2. Lundqvist C. Well-being in competitive sports—The feel-good factor? A review of conceptual considerations of well-being. Int Rev Sport Exerc Psychol. 2011;4:109–27.

3. Hong QN, Fàbregues S, Bartlett G, Boardman F, Cargo M, Dagenais P, et al. The Mixed Methods Appraisal Tool (MMAT) version 2018 for information professionals and researchers. Education for Information. 2018;34:285–91.
