# Supplementary material for: Psychological Interventions to Improve Elite Athlete Mental Wellbeing: A Systematic Review and Meta-analysis
Source: Sports Med. 2025 Jan 15;55(4):877–97. doi: 10.1007/s40279-024-02173-3 (PMC12011916; doi:10.1007/s40279-024-02173-3)
Supplement: Supplementary file 3 — Supplementary file3 (DOCX 59 KB) [file 40279_2024_2173_MOESM3_ESM.docx]

**Supplementary information. Online Resource 3.**

*Article:* Psychological Interventions to Improve Elite Athlete Mental Wellbeing: A Systematic Review and Meta-Analysis

*Journal:* Sports Medicine

*Authors:* Wei Wang, Matthew J. Schweickle, Emily Arnold, Stewart A Vella

*Corresponding author:* Wei Wang, School of Psychology, University of Wollongong, Wollongong, New South Wales, 2500, Australia. Email: ww862@uowmail.edu.au

**Search Strategies**

1. **Primary and updated search via databases**

| **Database** | **Search terms** | **Limiters** | **N (articles)** |
| --- | --- | --- | --- |
| Academic Search Complete (EBSCOhost)  2023-07-25  2024-05-03 | S1. TI ( wellbeing OR well- being OR wellness OR flourishing OR affect OR satisfaction OR happiness OR “mental health” ) OR AB ( wellbeing OR well-being OR wellness OR flourishing OR affect OR satisfaction OR happiness OR “mental health” )  S2. TI ( athlet* OR player* OR Para-athlet* ) OR AB ( athlet* OR player* OR Para-athlet* )  S3. TI ( intervention* OR program* OR workshop* OR control* OR trial* ) OR AB ( intervention* OR program* OR workshop* OR control* OR trial* )  S4. S1 AND S2 AND S3 | Full Text; Peer Reviewed; English | 1418 (2023-07-25)  92 (2024-05-03) |
| SPORTDiscus with Full Text  (EBSCOhost)  2023-07-25  2024-05-03 | S1. TI ( wellbeing OR well- being OR wellness OR flourishing OR affect OR satisfaction OR happiness OR “mental health” ) OR AB ( wellbeing OR well-being OR wellness OR flourishing OR affect OR satisfaction OR happiness OR “mental health” )  S2. TI ( athlet* OR player* OR Para-athlet* ) OR AB ( athlet* OR player* OR Para-athlet* )  S3. TI ( intervention* OR program* OR workshop* OR control* OR trial* ) OR AB ( intervention* OR program* OR workshop* OR control* OR trial* )  S4. S1 AND S2 AND S3 | Full Text; Peer Reviewed; English | 1360 (2023-07-25)  136 (2024-05-03) |
| MEDLINE with Full Text  (EBSCOhost)  2023-07-25  2024-05-03 | S1. TI ( wellbeing OR well- being OR wellness OR flourishing OR affect OR satisfaction OR happiness OR “mental health” ) OR AB ( wellbeing OR well-being OR wellness OR flourishing OR affect OR satisfaction OR happiness OR “mental health” )  S2. TI ( athlet* OR player* OR Para-athlet* ) OR AB ( athlet* OR player* OR Para-athlet* )  S3. TI ( intervention* OR program* OR workshop* OR control* OR trial* ) OR AB ( intervention* OR program* OR workshop* OR control* OR trial* )  S4. S1 AND S2 AND S3 | Full Text; Peer Reviewed; English | 1308 (2023-07-25)  100 (2024-05-03) |
| APA PsycArticles  (EBSCOhost)  2023-07-25  2024-05-03 | S1. TI ( wellbeing OR well- being OR wellness OR flourishing OR affect OR satisfaction OR happiness OR “mental health” ) OR AB ( wellbeing OR well-being OR wellness OR flourishing OR affect OR satisfaction OR happiness OR “mental health” )  S2. TI ( athlet* OR player* OR Para-athlet* ) OR AB ( athlet* OR player* OR Para-athlet* )  S3. TI ( intervention* OR program* OR workshop* OR control* OR trial* ) OR AB ( intervention* OR program* OR workshop* OR control* OR trial* )  S4. S1 AND S2 AND S3 | Full Text; Peer Reviewed; English | 76 (2023-07-25)  25 (2024-05-03) |
| APA PsycInfo  (EBSCOhost)  2023-07-25  2024-05-03 | S1. TI ( wellbeing OR well- being OR wellness OR flourishing OR affect OR satisfaction OR happiness OR “mental health” ) OR AB ( wellbeing OR well-being OR wellness OR flourishing OR affect OR satisfaction OR happiness OR “mental health” )  S2. TI ( athlet* OR player* OR Para-athlet* ) OR AB ( athlet* OR player* OR Para-athlet* )  S3. TI ( intervention* OR program* OR workshop* OR control* OR trial* ) OR AB ( intervention* OR program* OR workshop* OR control* OR trial* )  S4. S1 AND S2 AND S3 | Full Text; Peer Reviewed; English | 560 (2023-07-25)  128 (2024-05-03) |
| Google Scholar  2023-08-03  2024-05-03 | Intervention, Wellbeing, Athletes | First 200 results only, sort by relevance | 200 (2023-07-25)  200 (2024-05-03) |
| ProQuest Dissertations & Theses Global  2023-09-19  2024-05-03 | S1. TI ( wellbeing OR well- being OR wellness OR flourishing OR affect OR satisfaction OR happiness OR “mental health” ) OR AB ( wellbeing OR well-being OR wellness OR flourishing OR affect OR satisfaction OR happiness OR “mental health” )  S2. TI ( athlet* OR player* OR Para-athlet* )  S3. TI ( intervention* OR program* OR workshop* OR control* OR trial* )  S4. S1 AND S2 AND S3 | Full Text; English | 136 (2023-07-25)  1 (2024-05-03) |
| **Total** |  |  | **5058 (2023-07-25)**  **682 (2024-05-03)** |

1. **Primary and updated search via other methods: five systematic reviews and one scoping review**

| **Author (s)** | **Year** | **Title** | **N (Studies)** |
| --- | --- | --- | --- |
| Breslin et al. [1] | 2022 | An updated systematic review of interventions to increase awareness of mental health and well-being in athletes, coaches, officials and parents | 28 |
| Delfin et al. [2] | 2022 | Mental Health Interventions for Elite-Level Athletes in a Sport-Specific Context: A Systematic Review | 9 |
| Grilli et al. [3] | 2021 | Effects of Yoga Interventions on Psychological Health and Performance of Competitive Athletes: A Systematic Review | 6 |
| Myall et al. [4] | 2022 | Effect of mindfulness-based programmes on elite athlete mental health: A systematic review and meta-analysis | 12 |
| Tierney et al. [5] | 2020 | A Systematic Review of Mindfulness Interventions’ Impact on Athlete Emotional Distress & Wellbeing | 13 |
| Ekelund et al. [6] | 2023 | Interventions for improving mental health in athletes: a scoping review | 44 |
| **Total** |  |  | **112** |

**References:**

1. Breslin G, Shannon S, Cummings M, Leavey G. An updated systematic review of interventions to increase awareness of mental health and well-being in athletes, coaches, officials and parents. Syst Rev. 2022;11:99.

2. Delfin D, Gray H, Wilkerson AH. Mental Health Interventions for Elite-Level Athletes in a Sport-Specific Context: A Systematic Review. Am J Health Educ. 2022;53:297–311.

3. Grilli Cadieux E, Gemme C, Dupuis G. Effects of Yoga Interventions on Psychological Health and Performance of Competitive Athletes: A Systematic Review. Journal of Science in Sport and Exercise. 2021;3:158–66.

4. Myall K, Montero-Marin J, Gorczynski P, Kajee N, Syed Sheriff R, Bernard R, et al. Effect of mindfulness-based programmes on elite athlete mental health: a systematic review and meta-analysis. Br J Sports Med. 2023;57:99–108.

5. Tierney TN. A Systematic Review of Mindfulness Interventions’ Impact on Athlete Emotional Distress & Wellbeing [Master’s thesis]. Theses and Dissertations-Kinesiology and Health Promotion: University of Kentucky; 2020.

6. Ekelund R, Holmström S, Gustafsson H, Ivarsson A, Lundqvist C, Stenling A. Interventions for improving mental health in athletes: a scoping review. Int Rev Sport Exerc Psychol. 2023;1–36.
