# Supplementary material for: Psychological Interventions to Improve Elite Athlete Mental Wellbeing: A Systematic Review and Meta-analysis
Source: Sports Med. 2025 Jan 15;55(4):877–97. doi: 10.1007/s40279-024-02173-3 (PMC12011916; doi:10.1007/s40279-024-02173-3)
Supplement: Supplementary file 4 — Supplementary file4 (DOCX 33 KB) [file 40279_2024_2173_MOESM4_ESM.docx]

**Supplementary information. Online Resource 4.**

*Article:* Psychological Interventions to Improve Elite Athlete Mental Wellbeing: A Systematic Review and Meta-Analysis

*Journal:* Sports Medicine

*Authors:* Wei Wang, Matthew J. Schweickle, Emily Arnold, Stewart A Vella

*Corresponding author:* Wei Wang, School of Psychology, University of Wollongong, Wollongong, New South Wales, 2500, Australia. Email: ww862@uowmail.edu.au

**Full-text screening tool**

|  | Yes | No |
| --- | --- | --- |
| **Full text**  Does the paper have full-text access? |  |  |
|  |  | Exclude |
| **Language**  Is the full paper in English? |  |  |
|  |  | Exclude |
| **Type of study**  Is the study described as one of the following: |  |  |
| 1. Randomised controlled trial |  |  |
| 1. Non-randomised controlled trial |  |  |
| 1. Pre- and post-test study |  |  |
| 1. Qualitative study |  |  |
|  |  | Exclude |
| **Type of interventions**  Is the study a psychological intervention? |  |  |
|  |  | Exclude |
| **Outcomes**  Does the study report the outcomes (quantitative/qualitative) as one of the following: |  |  |
| 1. Emotional wellbeing |  |  |
| 1. Psychological wellbeing |  |  |
| 1. Social wellbeing |  |  |
| 1. Integrated wellbeing |  |  |
|  |  | Exclude |
| **Participants**  Are the participants elite athletes (university elite, national, international, and/or Olympic levels)? Excluding young athletes (< 18), retired athletes, and mixed samples. |  |  |
|  |  |  |
| Include for follow up |  |  |
