# Supplementary material for: Psychological Interventions to Improve Elite Athlete Mental Wellbeing: A Systematic Review and Meta-analysis
Source: Sports Med. 2025 Jan 15;55(4):877–97. doi: 10.1007/s40279-024-02173-3 (PMC12011916; doi:10.1007/s40279-024-02173-3)
Supplement: Supplementary file 5 — Supplementary file5 (DOCX 37 KB) [file 40279_2024_2173_MOESM5_ESM.docx]

**Supplementary information. Online Resource 5.**

*Article:* Psychological Interventions to Improve Elite Athlete Mental Wellbeing: A Systematic Review and Meta-Analysis

*Journal:* Sports Medicine

*Authors:* Wei Wang, Matthew J. Schweickle, Emily Arnold, Stewart A Vella

*Corresponding author:* Wei Wang, School of Psychology, University of Wollongong, Wollongong, New South Wales, 2500, Australia. Email: ww862@uowmail.edu.au

**A list of excluded studies during full-text eligibility assessment (n = 121)**

| **Not wellbeing outcomes^a^**  **(n = 58)** |  | A Brief Educational Intervention Using Acceptance and Commitment Therapy: Four Injured Athletes' Experiences |
| --- | --- | --- |
|  |  | A brief mindfulness and yoga intervention with an entire NCAA Division I athletic team: An initial investigation |
|  |  | A brief online video-based intervention to promote mental health help-seeking in the context of injuries for athletes: A pilot study |
|  |  | A Combined Sleep Hygiene and Mindfulness Intervention to Improve Sleep and Well-Being During High-Performance Youth Tennis Tournaments |
|  |  | A mindfulness intervention for collegiate athletes with concussion |
|  |  | A pilot study of a mindfulness-based program (MBSoccerP): The potential role of mindfulness, self-compassion and psychological flexibility on flow and elite performance in soccer athletes |
|  |  | A Program to Reduce Stigma Toward Mental Illness and Promote Mental Health Literacy and Help-Seeking in National Collegiate Athletic Association Division I Student-Athletes |
|  |  | A smartphone enabled slow-paced breathing intervention in dual career athletes |
|  |  | Academic Course Combining Psychological Skills Training and Life Skills Education for University Students and Student-Athletes |
|  |  | An evaluation of a health, wellbeing and lifestyle promotion intervention among a cohort of Year 1 male Gaelic Games student athletes in an Irish University setting |
|  |  | An individualized multimodal mental skills intervention for college athletes undergoing injury rehabilitation athletes undergoing injury rehabilitation |
|  |  | An individualized multimodal mental skills intervention for college athletes undergoing injury rehabilitation |
|  |  | Applying self-compassion in sport: An intervention with women athletes |
|  |  | Athletes Connected: Results From a Pilot Project to Address Knowledge and Attitudes About Mental Health Among College Student-Athletes |
|  |  | Attitudes toward Help-Seeking and Mental Health among College Athletes: Impact of a Psycho-educational Workshop |
|  |  | CMCL: An Online Resilience Training Program for College Athletes |
|  |  | Coach Encouragement During Soccer Practices Can Influence Players’ Mental and Physical Loads |
|  |  | Cognitive-Affective Stress Management Training With High Performance Youth Volleyball Players: Effects on Affect, Cognition, and Performance |
|  |  | Developing Performance Using Rational Emotive Behavior Therapy (REBT): A Case Study with an Elite Archer |
|  |  | Developing self-determined motivation and performance with an Elite Athlete: integrating motivational interviewing with rational emotive behavior therapy |
|  |  | Effect of yoga program on mental health: competitive anxiety in semarang badminton athletes |
|  |  | Effects of a formal goal setting program on recovery after athletic injury |
|  |  | Effects of a mindfulness intervention on sports‐anxiety, pessimism, and flow in competitive cyclists |
|  |  | Effects of mindfulness-acceptance-commitment (MAC) on sport-specific dispositional mindfulness, emotion regulation, and self-rated athletic performance in a multiple-sport population: an RCT study |
|  |  | Emotional competencies training for tennis players: Effectiveness of an online individualised psychological support program |
|  |  | Evaluation of the Online Sport-Specific Mindfulemotions Program in a Population of Scandinavian Elite Athletes Measuring Psychological Parameters |
|  |  | Examining the Effects of Mindfulness Training on Stress and Anxiety in Sport |
|  |  | Examining the effects of mindfulness–acceptance–commitment training on self-compassion and grit among elite female athletes |
|  |  | Examining the effects of rational emotive behavior therapy on performance outcomes in elite paralympic athletes |
|  |  | How does a sport psychological intervention help professional cyclists to cope with their mental health during the COVID-19 lockdown? |
|  |  | Impact of comprehensive athlete-centric nursing using the information-motivation-behaviour skills model and mind mapping on self-care and recovery in athletes undergoing cancer treatment |
|  |  | Influences of physical, environment, task, timing, learning, emotion, perspective (PETTLEP) intervention on psychological resilience, psychological skills, anxiety and depression of athletes |
|  |  | Intervention study using a leaflet entitled 'three benefits of 'go to bed early! get up early! and intake nutritionally rich breakfast!' a message for athletes' to improve the soccer performance of university soccer team |
|  |  | Mental Health Prevalence and Biofeedback Intervention for Student-Athletes |
|  |  | Mental Health Referral for Student-Athletes: Web-Based Education and Training |
|  |  | Mental Toughness Training Intervention for Collegiate Track and Field Athletes |
|  |  | Mindful compassion training on elite soccer: Effects, roles and associations on flow, psychological distress and thought suppression |
|  |  | Mindful Recovery: A Case Study of a Burned-Out Elite Shooter |
|  |  | Mindfulness training and health-related quality of life in male NCAA division I athletes |
|  |  | Pilot trial of an online sexual violence prevention program for college athletes |
|  |  | Psycholinguistic Changes in Athletes' Grief Response to Injury After Written Emotional Disclosure |
|  |  | Psychological Intervention Based on Mental Relaxation to Manage Stress in Female Junior Elite Soccer Team: Improvement in Cardiac Autonomic Control, Perception of Stress and Overall Health |
|  |  | Reducing eating disorder risk among male athletes: A randomized controlled trial investigating the male athlete body project. |
|  |  | Reducing the Risk of Disordered Eating Among Female Athletes: A Test of Alternative Interventions |
|  |  | Resilience in Collegiate Student-Athletes: A Pilot Study of a Prevention and Resilience Training Program |
|  |  | "Short and Sweet": A Randomized Controlled Initial Investigation of Brief Online Psychological Interventions With Endurance Athletes |
|  |  | Stress-management interventions for female athletes: Relaxation and cognitive restructuring |
|  |  | Tackling Difficult Conversations: Student-Athletes, Mental Health, and Emerging Technology |
|  |  | Tackling Mental Health in Youth Sporting Programs: A Pilot Study of a Holistic Program |
|  |  | The adaptation and evaluation of a pilot mindfulness intervention promoting mental health in student athletes |
|  |  | The effect of mindfulness intervention on the psychological skills and shooting performances in male Collegiate basketball athletes in Macau: a quasi-experimental study |
|  |  | The effects from mindfulness training on Norwegian junior elite athletes in sport |
|  |  | The effects of a mindfulness-based program on the incidence of injuries in young male soccer players |
|  |  | The Effects of Feedback Valence and Style on Need Satisfaction, Self-Talk, and Perseverance Among Tennis Players: An Experimental Study |
|  |  | The effects of mindfulness training on mindfulness, anxiety, emotion dysregulation, and performance satisfaction among female student-athletes: The moderating role of age |
|  |  | The Effects of Mindfulness-Based Strategies on Perceived Stress and Psychobiosocial States in Athletes and Recreationally Active People |
|  |  | The impact of yoga-based interventions on jiu-jitsu wrestlers’ psychological profile: a pilot study |
|  |  | The mindfulness-based soccer program (MBSoccerP): Effects on elite athletes |
| **Not elite athletes or elite athletes < 18^b^**  **(n = 34)** |  | A Case Study Investigation Into a Group Online Sport Psychology Support Intervention for Injured Athletes |
|  |  | A Preliminary Investigation Into the Use of Counseling Skills in Support of Rehabilitation From Sport Injury |
|  |  | Cognitive-behavioral intervention effects on mood and cortisol during exercise training |
|  |  | Controlled Evaluation of an Optimization Approach to Mental Health and Sport Performance |
|  |  | Effect of brief mindfulness and relaxation inductions on anxiety, affect and brain activation in athletes |
|  |  | Effect of mindfulness based stress reduction (MBSR) in increasing pain tolerance and improving the mental health of injured athletes |
|  |  | Effects of a mental health intervention in athletes: Applying self-determination theory |
|  |  | Effects of a Motivational Self-Talk Intervention for Endurance Athletes Completing an Ultramarathon |
|  |  | Effects of a slow-paced breathing (SPB) intervention on young athletes' psychological adjustment: A study with female volleyball players |
|  |  | Effects of a slow-paced breathing (SPB) intervention on young athletes' psychological adjustment: A study with female volleyball players |
|  |  | Evaluating the Feasibility of a Self-Compassion Intervention for Competitive Men Athletes |
|  |  | Examining the effectiveness of an imagery intervention in enhancing athletes' eudaimonic well-being |
|  |  | Implementation and Evaluation of Mindfulness-Based Interventions to Improve Mental Health and Overall Wellbeing of College Student-Athletes |
|  |  | Implementation of a mindfulness-based stress reduction (MBSR) program to reduce stress, anxiety, and depression and to improve psychological well-being among retired Iranian football players |
|  |  | Influence of mindfulness on levels of impulsiveness, moods and pre-competition anxiety in athletes of different sports |
|  |  | Living your best life: The mindful pursuit of student-athlete thriving |
|  |  | Mindfulness in the sport academy classroom: Exploring benefits and barriers of a low-dose intervention |
|  |  | Mood lifters for college athletes: A biopsychosocial approach to improving mental health for student-athletes |
|  |  | Moving to Action: The Effects of a Self- Regulation Intervention on the Stress, Burnout, Well-Being, and Self-Regulation Capacity Levels of University Student-Athletes |
|  |  | One year follow-up of mindful sport performance enhancement (MSPE) with archers, golfers, and runners |
|  |  | Personal goal management intervention and mood states in soccer academies |
|  |  | Piloting a family-supported approach to concurrently optimize mental health and sport performance in athletes |
|  |  | Protecting and promoting the wellbeing of high-performance swimmers |
|  |  | Psychological skills training and perfectionism: A single-subject multiple baseline study |
|  |  | Putting imagery to good affect: A case study among youth swimmers |
|  |  | Resilience and Enhancement in Sport, Exercise, & Training (RESET): A brief self-compassion intervention with NCAA student-athletes |
|  |  | Sport psychological skills training and psychological well-being in youth athletes |
|  |  | The Adaptation and Evaluation of a Pilot Mindfulness Intervention Promoting Mental Health in Student Athletes |
|  |  | The Cognitive and Motivation Intervention Program in Youth Female Volleyball Players |
|  |  | The effect of short-term multidisciplinary intervention on the levels of anxiety, ego-resilience and affect in sports shooters with disabilities |
|  |  | The Effectiveness of a Brief Cognitive Intervention to Help Athletes Cope With Competition Loss |
|  |  | The evaluation of a psychological skills training programme for rugby players |
|  |  | The power of competence support: The impact of coaches and athlete leaders on intrinsic motivation and performance |
|  |  | Using Goal Setting to Enhance Positive Affect Among Junior Multievent Athletes |
| **Unclear data^c^**  **(n = 7)** |  | Does a Brief Mindfulness Intervention Improve Distress Tolerance Among Athletes? |
|  |  | Increasing Athlete Knowledge of Mental Health and Intentions to Seek Help: The State of Mind Ireland (SOMI) Pilot Program |
|  |  | Mindfulness practice is associated with improved wellbeing and reduced injury risk in female NCAA Division I athletes |
|  |  | Psycho-Pedagogical Prevention of Aggressive Behaviours in Athletes |
|  |  | The Athlete Gratitude Group (TAGG): Effects of coach participation in a positive psychology intervention with youth athletes |
|  |  | The effects of a self -esteem enhancement program on perceived performance among intercollegiate athletes |
|  |  | The effects of psychological skills training on mental toughness and psychological well-being of student-athletes |
| **Wrong study design^d^**  **(n = 12)** |  | A Mindfulness-and Acceptance-Based Intervention for Injured Athletes |
|  |  | A Strengths-Based Cognitive Behavioral Approach to Treating Depression and Building Resilience in Collegiate Athletics: The Individuation of an Identical Twin. |
|  |  | Case Study 4: A case study of cognitive behaviour therapy in tennis |
|  |  | Developing student-athlete school satisfaction and psychological well-being: The effects of academic psychological capital and engagement |
|  |  | Effectiveness Of Sport Psychology Interventions In Reducing Stress And Anxiety Among Competitive Athletes |
|  |  | Evaluation of a Division I Mid-Major University’s Student-Athlete Mental Health Program |
|  |  | Mindfulness and Regulatory Emotional Self-Efficacy of Injured Athletes Returning to Sports: The Mediating Role of Competitive State Anxiety and Athlete Burnout |
|  |  | Mindfulness in Collegiate Athletics: A Case Study at a Division I University |
|  |  | Preparing elite athletes for the career after the career: the functions of mentoring programmes |
|  |  | Psychological Effects of Fast- and Slow-Tempo Music Hayed during Volleyball Training in a National League Team |
|  |  | The Effectiveness of a Psychoeducational Program in Increasing Mental Health Knowledge in Collegiate Athletes: A Critically Appraised Topic |
|  |  | Using Cooperative Learning to Enhance the Academic and Social Experiences of Freshman Student Athletes |
| **No full text**  **(n = 4)** |  | Managing Athlete Perfectionism in Sport |
|  |  | Mind-Body Health Strategies for Supporting Student-Athletes. |
|  |  | Preliminary results of a sleep health intervention in student athletes: perceived changes to sleep, performance, and mental and physical well-being |
|  |  | The effects of group development techniques on a professional athletic team |
| **Not psychological interventions**  **(n = 4)** |  | An Integrated Mind–Body Training Intervention on an Elite Surfing Athlete: A Case Study |
|  |  | Feasibility of eyes open alpha power training for mental enhancement in elite gymnasts |
|  |  | Well-Being and Throwing Speed of Women Handball Players Affected by Feedback |
|  |  | Working Memory Training in Professional Football Players: A Small-Scale Descriptive Feasibility Study—The Importance of Personality, Psychological Well-Being, and Motivational Factors |
| **Not English^e^**  **(n = 2)** |  | Investigating the effectiveness of sports intervention based on intergenerational programs on happiness and mental well-being in bodybuilding athletes |
|  |  | The effect of positive psychological intervention program on mood state, self-esteem and happiness of university student athletes: Exploratory studies |

^a^Studies did not quantitatively or qualitatively report wellbeing outcomes defined in this review

^b^Studies recruited athletes who were not at the elite level or elite athletes who were below 18 years old

^c^Studies missed necessary data for eligibility assessment and their corresponding authors did not reply to our data request

^d^Studies were not intervention research or did not use the following methods: randomised controlled, quasi-experimental, qualitative, or mixed methods

^e^Studies did not provide English full text
