# Supplementary material for: Psychological Interventions to Improve Elite Athlete Mental Wellbeing: A Systematic Review and Meta-analysis
Source: Sports Med. 2025 Jan 15;55(4):877–97. doi: 10.1007/s40279-024-02173-3 (PMC12011916; doi:10.1007/s40279-024-02173-3)
Supplement: Supplementary file 6 — Supplementary file6 (DOCX 84 KB) [file 40279_2024_2173_MOESM6_ESM.docx]

**Supplementary information. Online Resource 6.**

*Article:* Psychological Interventions to Improve Elite Athlete Mental Wellbeing: A Systematic Review and Meta-Analysis

*Journal:* Sports Medicine

*Authors:* Wei Wang, Matthew J. Schweickle, Emily Arnold, Stewart A Vella

*Corresponding author:* Wei Wang, School of Psychology, University of Wollongong, Wollongong, New South Wales, 2500, Australia. Email: ww862@uowmail.edu.au

The following formulas were used to calculate individual studies’ SMD (*d*) and standard error (SE) [1].

$$d=\frac{\bar{X}_{1}-\bar{X}_{2}}{S_{within}}$$

$${SE}_{d}=\sqrt{V_{d}}$$

For studies with controlled groups (see table 1),‾*X_1_* and‾*X_2_* were the post-test means in the treatment and control groups; *S_within_* was the within-groups standard deviation, pooled across the two groups; *V_d_* was the variance of Cohen’s d; n_1_ and n_2_ were the sample sizes of the two groups; S_1_ and S_2_ were the post-test standard deviations in the two groups.

$$S_{within}=\sqrt{\frac{{(n}_{1}-1)S_{1}^{2}+{(n}_{2}-1)S_{2}^{2}}{n_{1}+n_{2}-2}}$$

$$V_{d}=\frac{n_{1}+n_{2}}{n_{1}n_{2}}+\frac{d^{2}}{2{(n}_{1}+n_{2})}$$

For studies without controlled groups (see table 2),‾*X_1_* and‾*X_2_* were the pre-test and post-test sample means; *S’_within_* was the within-group standard deviation; *S_diff_* was the standard deviation of the pretest-posttest difference; *r* was the pretest-posttest correlation; *V’_d_* was the variance of Cohen’s d; n was the sample size.

$${S'}_{within}=\frac{S_{diff}}{\sqrt{2(1-r)}}$$

$${V'}_{d}=\left( \frac{1}{n}+\frac{d^{2}}{2n} \right)2(1-r)$$

| Name | $\bar{X}_{1}$ | S_1_ | n_1_ | $\bar{X}_{2}$ | S_1_ | n_2_ | Swithin | **d (SMD)** | V_d_ | **SE_d_** |
| --- | --- | --- | --- | --- | --- | --- | --- | --- | --- | --- |
| Ajilchi et al. [2] | 80.62 | 9.31 | 21 | 72.09 | 9.27 | 21 | 9.29 | **0.92** | 0.11 | **0.32** |
| Baltzell & Akhtar [3] | 32.21 | 7.47 | 19 | 33.56 | 6.05 | 23 | 6.73 | **-0.20** | 0.10 | **0.31** |
| Brent [4] | 37.94 | 4.85 | 16 | 36.64 | 5.58 | 14 | 5.20 | **0.25** | 0.13 | **0.37** |
| Johnson [5] | 3.49 | 0.32 | 14 | 3.08 | 0.43 | 44 | 0.41 | **1.01** | 0.10 | **0.32** |
| Jones et al. [6] | 192.36 | 35.18 | 14 | 164.08 | 18.44 | 11 | 29.11 | **0.97** | 0.18 | **0.43** |
| Laureano et al. [7] | 39.8 | 4.92 | 20 | 38.67 | 3.92 | 21 | 4.44 | **0.25** | 0.10 | **0.31** |
| Macdougall et al. [8] (Psychological wellbeing) | 214.22 | 22.44 | 9 | 204 | 26.31 | 9 | 24.45 | **0.42** | 0.23 | **0.48** |
| Macdougall et al. [8] (Positive affect) | 25.3 | 4.2 | 9 | 21.1 | 4.4 | 9 | 4.30 | **0.98** | 0.25 | **0.50** |
| Macdougall et al. [8] (Satisfaction with life) | 29 | 5.4 | 9 | 23 | 6 | 9 | 5.71 | **1.05** | 0.25 | **0.50** |
| Macdougall et al. [8] (Social wellbeing) | 76.56 | 9.17 | 9 | 80.56 | 11.08 | 9 | 10.17 | **-0.39** | 0.23 | **0.48** |
| Podlog et al. [9] | 4.28 | 0.23 | 8 | 3.54 | 0.29 | 8 | 0.26 | **2.83** | 0.50 | **0.71** |
| Rooks et al. [10] | 35.76 | 9.21 | 56 | 35.91 | 6.74 | 44 | 8.22 | **-0.02** | 0.04 | **0.20** |

**Table 1** Studies with controlled groups

**Table 2** Studies without controlled groups

| Name | $\bar{X}_{1}-\bar{X}_{2}$ | Sdiff | n | r | S'within | **d (SMD)** | V'_d_ | **SE_d_** |
| --- | --- | --- | --- | --- | --- | --- | --- | --- |
| Fallon [11] | 0.74 | 5.16 | 7 | 0.48 | 5.06 | **0.15** | 0.15 | **0.39** |
| Gabana et al. [12] | 0.47 | 0.74 | 51 | 0.52 | 0.76 | **0.62** | 0.02 | **0.15** |
| Laslett & Uphill [13] | 0.4 | 3.71 | 3 | 0.97 | 15.15 | **0.03** | 0.02 | **0.14** |
| Micoogullari & Ekmekci [14] | 14.86 | 21.63 | 26 | 0.55 | 22.80 | **0.65** | 0.04 | **0.20** |
| Moesch et al. [15] | 14.3 | 12.22 | 6 | 0.74 | 16.95 | **0.84** | 0.12 | **0.34** |
| Stellefson et al. [16] | 3.28 | 11.08 | 10 | 0.34 | 9.64 | **0.34** | 0.14 | **0.37** |

**References:**

1. Borenstein M, Hedges L V., Higgins JPT, Rothstein HR. Introduction to Meta‐Analysis. Wiley; 2009.

2. Ajilchi B, Mohebi M, Zarei S, Kisely S. Effect of a mindfulness programme training on mental toughness and psychological well-being of female athletes. Australasian Psychiatry. 2022;30:352–6.

3. Baltzell A, Akhtar VL. Mindfulness meditation training for sport (MMTS) intervention: Impact of MMTS with division I female athletes. The Journal of Happiness & Well-Being. 2014;2:160–73.

4. Brent ME. A cognitive-behavioral stress management intervention for division I collegiate student-athletes [Doctoral dissertation]. ProQuest Dissertations and Theses Global: Ohio State University; 2004.

5. Johnson U. Short-Term Psychological Intervention: A Study of Long-Term-injured Competitive Athletes. J Sport Rehabil. 2000;9:207–18.

6. Jones BJ, Kaur S, Miller M, Spencer RMC. Mindfulness-Based Stress Reduction Benefits Psychological Well-Being, Sleep Quality, and Athletic Performance in Female Collegiate Rowers. Front Psychol. 2020;11.

7. Laureano C, Grobbelaar HW, Nienaber AW. Facilitating the coping self-efficacy and psychological well-being of student rugby players. South African Journal of Psychology. 2014;44:483–97.

8. Macdougall H, O’Halloran P, Sherry E, Shields N. A Pilot Randomised Controlled Trial to Enhance Well-Being and Performance of Athletes in Para Sports. European Journal of Adapted Physical Activity. 2019;12:7–7.

9. Podlog LW, Heil J, Burns RD, Bergeson S, Iriye T, Fawver B, et al. A Cognitive Behavioral Intervention for College Athletes With Injuries. Sport Psychol. 2020;34:111–21.

10. Rooks JD, Morrison AB, Goolsarran M, Rogers SL, Jha AP. “We Are Talking About Practice”: the Influence of Mindfulness vs. Relaxation Training on Athletes’ Attention and Well-Being over High-Demand Intervals. Journal of Cognitive Enhancement. 2017;1:141–53.

11. Fallon J. Yoga as an intervention for stress reduction and enhanced wellbeing in African American athletes [Doctoral dissertation]. ProQuest Dissertations and Theses Global: Utah State University; 2008.

12. Gabana NT, Steinfeldt J, Wong YJ, Chung YB, Svetina D. Attitude of Gratitude: Exploring the Implementation of a Gratitude Intervention with College Athletes. J Appl Sport Psychol. 2019;31:273–84.

13. Laslett B, Uphill M. An Online Intervention to Support Student-Athlete Mental Health: Implementation, Evaluation, and Critical Reflection. Case Studies in Sport and Exercise Psychology. 2020;4:S1-54-S1-61.

14. Miçooğullari BO, Ekmekçi R. Evaluation of a Psychological Skill Training Program on Mental Toughness and Psychological Wellbeing for Professional Soccer Players. Universal Journal of Educational Research. 2017;5:2312–9.

15. Moesch K, Ivarsson A, Johnson U. “Be Mindful Even Though It Hurts”: A Single-Case Study Testing the Effects of a Mindfulness- and Acceptance-Based Intervention on Injured Athletes’ Mental Health. J Clin Sport Psychol. 2020;14:399–421.

16. Stellefson M, Bopp T, Odio M, Wang MQ, Zhang L. Development of the Life After Sports Transition (LAST) Online Course for Collegiate Student-Athletes: Pretest-Posttest Study. Journal of Athlete Development and Experience. 2020;2.
