# Supplementary material for: Psychological Interventions to Improve Elite Athlete Mental Wellbeing: A Systematic Review and Meta-analysis
Source: Sports Med. 2025 Jan 15;55(4):877–97. doi: 10.1007/s40279-024-02173-3 (PMC12011916; doi:10.1007/s40279-024-02173-3)
Supplement: Supplementary file 7 — Supplementary file7 (DOCX 113 KB) [file 40279_2024_2173_MOESM7_ESM.docx]

**Supplementary information. Online Resource 7.**

*Article:* Psychological Interventions to Improve Elite Athlete Mental Wellbeing: A Systematic Review and Meta-Analysis

*Journal:* Sports Medicine

*Authors:* Wei Wang, Matthew J. Schweickle, Emily Arnold, Stewart A Vella

*Corresponding author:* Wei Wang, School of Psychology, University of Wollongong, Wollongong, New South Wales, 2500, Australia. Email: ww862@uowmail.edu.au

**Attributive statements regarding facilitators and barriers to implementation**

| **Domain** | **Construct** | **Facilitator** |
| --- | --- | --- |
| **Intervention characteristics** | **Adaptability** | - **Using language accessible to athletes**   “The results from this study indicate that it may be valuable to frame self-compassion as a catalyst of courage. Several participants indicated that this language used throughout the intervention to introduce and explain self-compassion was vital to their receptivity and skill acquisition” ([1], p. 209)   - **Incorporating sport psychological elements**   “MMTS included using imagery (of aversive moments in sport that cause negative emotions) and guided self-talk cue words (i.e. positive affirmations were used; labeling emotions when they occurred, such as “there is fear”.) Though these cues were used differently than traditional PST, using such alternative cues (compared to traditional mindfulness mediation practice) paired with acceptance and non-judgment may be of use to sport psychology practitioners using mindfulness-based interventions” ([2], p.169)  “Combining the themes of mental skills for performance and mental health in each session may help to get the buy-in of athletes, increase participation, and decrease stigma toward mental health interventions among athletes” ([3], p. 15)   - **Shortening duration of each session**   “The class session duration was shortened from the standard 150 min to promote high attendance and more time for self-practice” ([4], p. 4)   - **Applicability to sport and life**   “The positive shift in attitude coincided with the introduction of active, directive meditation exercises (e.g., concentration ladders), and mental practice being specifically connected to soccer” ([5], p. 240)  “On a surprising note, the athlete participants not only perceived the program to be beneficial to their performance on the court but also expressed positive gains in their academic and personal development” ([1], p. 210)  “With regard to the BEVS, participants took 30–90 min to complete the task and reported being satisfied with the effects manifested by the intervention on their mental health and well-being, and finding it very useful…It has also given me time to take a look at exactly where I want to go with different areas of my life and how I want to progress” ([6], p. 58)  “I really like this practice because not only did it help me on the court but it helped me off the court, in my everyday life” ([7], p. 154)  “All players expressed that I-ACT met their needs and was relevant to them as ice hockey players…All players said that I-ACT was helpful in life outside of sports” ([8], p. 6-7)  “Participant H noted helping him focus on tasks relevant for academic and athletic performance as a benefit of mindfulness practice” ([9], p. 64) |
| **Individual characteristics** | **Personal attributes** | - **Instructor’ capacity to connect with athletes**   “The athletes’ connection (buy-in) to the meditation program may be partly attributable to the meditation leader. First, three athletes reported that they liked the meditation facilitator’s personality (“I really like [the facilitator], I thought that he was a pretty good personality for our team.”) And, second, the facilitator attending a game was pivotal” ([5], p. 233)  “The athletes also noted that the instructor did a great job balancing listening, answering questions, and keeping them engaged with mental training activities” ([1], p. 206)  “The trust and rapport established with both teams was significant to the athletes’ interest and commitment to the positive exercises” ([10], p. 179)  “All players said it was positive and helpful with the chat function with the psychologist and that quick answers were provided when needed” ([8], p. 6) |
| **Inner setting** | **Readiness for implementation** | - **Coach support**   “Given coach participation, including strong buy-in from the coaches and possibly more attention by the athletes to the process due to coach presence, the athletes could have gained more from the program” ([5], p. 240)  “I think our coaches came to some of our meetings, and I think they’ve also kind of realized that those things help us and make us want to keep working hard and it’s, like, doing those little things definitely helps” ([10], p. 112)   - **Available resources**   “In-class instruction was supplemented with handouts, workbooks, and audio recordings for in-home practice” ([4], p. 4) |
|  | **Implementation climate** | - **Relaxing learning climate**   “Four of the nine participants described the time in the program as a resource to slow down and temporarily put a pause on their athletic, academic, social, and personal responsibilities” ([1], p. 207) |
| **Process** | **Engaging** | - **Mutiple in-session activities**   “The participant considered the activities helpful and suggested that even more activities could be used” ([11], p. 30)  “All athletes thought it was positive that the intervention was delivered in different media formats (text, audio exercises, and videos)” ([8], p. 5)   - **Between-session assignments**   “The participant considered the homework helpful and inquired about not having homework in one of the weeks”([11], p. 30)   - **Teammate support**   “That’s what I really liked about it. I liked that the team was doing the same thing” ([5], p. 238)  “Team consultation is still a valuable aspect of sport psychology work, as it is time effective and provides athletes with opportunities to learn from each other and obtain social support” ([12], p. 80)  “Given the increase in perceived social support immediately following the gratitude workshop and the magnitude of the effect, a gratitude-focused team session may have the potential to improve one’s view of teammates and/or coaches” ([13], p. 281)  “I am from the south and no one else is from my state but hearing everyone’s experiences helped me open up, we all got problems and stuff back home but this class brings everyone together” ([14], p. 275)  “H noted that participation in this course contributed to his building ‘friendships’ and developing ‘bonds’” ([9], p. 63) |

| **Domain** | **Construct** | **Barrier** |
| --- | --- | --- |
| **Intervention characteristics** | **Complexity** | - **Complex and unfamiliar intervention contents**   “Many interviewees reported feeling “bad” at meditation” ([5], p. 239)  “Athletes reported that the progressive muscle relaxation was difficult to practice on their own. Doing PMR was a challenge without the facilitator's calming voice guiding them through the steps” ([12], p. 76)  “Some of the student-athletes found the mindfulness and meditative practices delivered in the sessions uncomfortable and frustrating” ([1], p. 210)  “The only challenges identified by some of the athletes was the difficulty of implementing the practice at certain times, especially with regards to keeping one’s mind focused and away from wandering” ([7], p. 156)  “He also described difficulties understanding the juggling-exercise (defusion)” ([8], p. 6) |
| **Process** | **Executing** | - **Athletes’ busy schedules**   “I’m at a point right now where my school and work is very hectic and I just feel like I don’t have time, honestly. Even though it is a couple minutes, I just feel overwhelmed with stuff” ([1], p. 208)  “Participants received only 6 weeks of yoga classes, half of these or more being only one class per week due to unanticipated schedule conflicts with athletic demands” ([15], p. 69-70)  “Another important factor to discuss is that one of the teams that was in season at the time found this intervention difficult to complete. The team had extra challenges during that specific season because of changes of game schedule, but it is still noteworthy that such robust intervention may be better suited for the off-season period” ([3], p. 15)  “In addition, due to the aforementioned active basketball season, the sessions were not evenly spaced during the treatment period as adjustments had to be made to account for the schedule” ([7], p.157) |
|  | **Reflecting and evaluating** | - **Risk of bias**   “Only 9 of the 23 total participants voluntarily agreed to complete the interview portion of the MMTS 2.0 program. These nine participants might have gotten more out of the program, indicating a potential bias toward a more positive experience of the program and findings only applicable to these nine athletes” ([1], p. 210) |

**References:**

1. Cote T, Baltzell A, Diehl R. A Qualitative Exploration of Division I Tennis Players Completing the Mindfulness Meditation Training for Sport 2.0 Program. Sport Psychol. 2019;33:203–12.

2. Baltzell A, Akhtar VL. Mindfulness meditation training for sport (MMTS) intervention: Impact of MMTS with division I female athletes. The Journal of Happiness & Well-Being. 2014;2:160–73.

3. Fogaca JL. Combining Mental Health and Performance Interventions: Coping and Social Support for Student-Athletes. J Appl Sport Psychol. 2021;33:4–19.

4. Jones BJ, Kaur S, Miller M, Spencer RMC. Mindfulness-Based Stress Reduction Benefits Psychological Well-Being, Sleep Quality, and Athletic Performance in Female Collegiate Rowers. Front Psychol. 2020;11.

5. Baltzell A, Caraballo N, Chipman K, Hayden L. A Qualitative Study of the Mindfulness Meditation Training for Sport: Division I Female Soccer Players’ Experience. J Clin Sport Psychol. 2014;8:221–44.

6. Laslett B, Uphill M. An Online Intervention to Support Student-Athlete Mental Health: Implementation, Evaluation, and Critical Reflection. Case Studies in Sport and Exercise Psychology. 2020;4:S1-54-S1-61.

7. Vidic Z, St. Martin M, Oxhandler R. Mindfulness Intervention With a U.S. Women’s NCAA Division I Basketball Team: Impact on Stress, Athletic Coping Skills and Perceptions of Intervention. Sport Psychol. 2017;31:147–59.

8. Reinebo G, Björverud LG, Parling T, Andersson G, Jansson-Fröjmark M, Lundgren T. Development and experiences of an internet-based acceptance and commitment training (I-ACT) intervention in ice hockey players: a qualitative feasibility study. Front Sports Act Living. 2024;6.

9. Leap P. Adversity, Resilience, Mental Health, and Well-being in First-Year Student-Athletes: A Mixed Methods Intervention Study [Doctoral dissertation]. ProQuest Dissertations and Theses Global: James Madison University; 2023.

10. Morton S. A win at all’values’ mentality: a phenomenological investigation of lived experiences of college gymnasts and volleyball players in a comprehensive positive psychology intervention [Doctoral dissertation]. ProQuest Dissertations and Theses Global: University of Missouri; 2014.

11. Green J. Effects of a Purpose Intervention on Purpose and Well-Being Outcomes in Elite Athletes: A Case Study [Master’s thesis]. ProQuest Dissertations and Theses Global: California State University; 2022.

12. Brent ME. A cognitive-behavioral stress management intervention for division I collegiate student-athletes [Doctoral dissertation]. ProQuest Dissertations and Theses Global: Ohio State University; 2004.

13. Gabana NT, Steinfeldt J, Wong YJ, Chung YB, Svetina D. Attitude of Gratitude: Exploring the Implementation of a Gratitude Intervention with College Athletes. J Appl Sport Psychol. 2019;31:273–84.

14. Chandler GE, Kalmakis KA, Chiodo L, Helling J. The Efficacy of a Resilience Intervention Among Diverse, At-Risk, College Athletes: A Mixed-Methods Study. J Am Psychiatr Nurses Assoc. 2020;26:269–81.

15. Fallon J. Yoga as an intervention for stress reduction and enhanced wellbeing in African American athletes [Doctoral dissertation]. ProQuest Dissertations and Theses Global: Utah State University; 2008.
