# Supplementary material for: Psychological Interventions to Improve Elite Athlete Mental Wellbeing: A Systematic Review and Meta-analysis
Source: Sports Med. 2025 Jan 15;55(4):877–97. doi: 10.1007/s40279-024-02173-3 (PMC12011916; doi:10.1007/s40279-024-02173-3)
Supplement: Supplementary file 8 — Supplementary file8 (DOCX 38 KB) [file 40279_2024_2173_MOESM8_ESM.docx]

**Supplementary information. Online Resource 8.**

*Article:* Psychological Interventions to Improve Elite Athlete Mental Wellbeing: A Systematic Review and Meta-Analysis

*Journal:* Sports Medicine

*Authors:* Wei Wang, Matthew J. Schweickle, Emily Arnold, Stewart A Vella

*Corresponding author:* Wei Wang, School of Psychology, University of Wollongong, Wollongong, New South Wales, 2500, Australia. Email: ww862@uowmail.edu.au

| Yes | No | Can’t tell |
| --- | --- | --- |

**Critical appraisal: Mixed Methods Appraisal Tools** [1]

| 1. Qualitative | 1.1. Is the qualitative approach appropriate to answer the research question? | 1.2. Are the qualitative data collection methods adequate to address the research question? | 1.3. Are the findings adequately derived from the data? | 1.4. Is the interpretation of results sufficiently substantiated by data? | 1.5. Is there coherence between qualitative data sources, collection, analysis and interpretation? | Overall quality of study |
| --- | --- | --- | --- | --- | --- | --- |
| Baltzell et al. (2014) |  |  |  |  |  | 80% |
| Cote et al. (2019) |  |  |  |  |  | 80% |
| Morton (2014) |  |  |  |  |  | 80% |
| Reinebo et al. (2024) |  |  |  |  |  | 100% |
|  |  |  |  |  |  |  |
| 2. Quantitative  randomised controlled  trials | 2.1. Is randomisation appropriately performed? | 2.2. Are the groups comparable at baseline? | 2.3. Are there complete outcome data? | 2.4. Are outcome assessors blinded to the intervention provided? | 2.5 Did the participants adhere to the assigned intervention? | Overall quality of study |
| Ajilchi, et al. (2022) |  |  |  |  |  | 40% |
| Johnson (2000) |  |  |  |  |  | 20% |
| Macdougall et al. (2019) |  |  |  |  |  | 60% |
| Rooks et al. (2017) |  |  |  |  |  | 20% |
|  |  |  |  |  |  |  |
| 3. Quantitative non-randomised (Quasi-experimental) | 3.1. Are the participants representative of the target population? | 3.2. Are measurements appropriate regarding both the outcome and intervention (or exposure)? | 3.3. Are there complete outcome data? | 3.4. Are the confounders accounted for in the design and analysis? | 3.5. During the study period, is the intervention administered (or exposure occurred) as intended? | Overall quality of study |
| Baltzell and Akhtar (2014) |  |  |  |  |  | 40% |
| Brent (2004) |  |  |  |  |  | 60% |
| Fallon (2008) |  |  |  |  |  | 40% |
| Fogaca (2021) |  |  |  |  |  | 60% |
| Gabana et al. (2019) |  |  |  |  |  | 60% |
| Gavrilova (2016) |  |  |  |  |  | 80% |
| Jones et al. (2020) |  |  |  |  |  | 20% |
| Laslett and Uphill (2020) |  |  |  |  |  | 40% |
| Laureano et al. (2014) |  |  |  |  |  | 60% |
| Miçoogullari and Ekmekçi (2017) |  |  |  |  |  | 60% |
| Moesch et al. (2020) |  |  |  |  |  | 60% |
| Podlog et al. (2020) |  |  |  |  |  | 60% |
| Stellefson et al. (2020) |  |  |  |  |  | 60% |
| Van Ryswyk et al. (2017) |  |  |  |  |  | 20% |
|  |  |  |  |  |  |  |
| 4. Mixed methods | 4.1. Is there an adequate rationale for using a mixed methods design to address the research question? | 4.2. Are the different components of the study effectively integrated to answer the research question? | 4.3. Are the outputs of the integration of qualitative and quantitative components adequately interpreted? | 4.4. Are divergences and inconsistencies between quantitative and qualitative results adequately addressed? | 4.5. Do the different components of the study adhere to the quality criteria of each tradition of the methods involved? | Overall quality of study |
| Chandler et al. (2020) |  |  |  |  |  | 60% |
| Green (2022) |  |  |  |  |  | 60% |
| Leap (2023) |  |  |  |  |  | 40% |
| Poulus et al. (2023) |  |  |  |  |  | 80% |
| Vidic et al. (2017) |  |  |  |  |  | 60% |

**References:**

1. Hong QN, Fàbregues S, Bartlett G, Boardman F, Cargo M, Dagenais P, et al. The Mixed Methods Appraisal Tool (MMAT) version 2018 for information professionals and researchers. Education for Information. 2018;34:285–91.
